# The Increased Activity of TRPV4 Channel in the Astrocytes of the Adult Rat Hippocampus after Cerebral Hypoxia/Ischemia

**DOI:** 10.1371/journal.pone.0039959

**Published:** 2012-06-27

**Authors:** Olena Butenko, David Dzamba, Jana Benesova, Pavel Honsa, Valentina Benfenati, Vendula Rusnakova, Stefano Ferroni, Miroslava Anderova

**Affiliations:** 1 Department of Cellular Neurophysiology, Institute of Experimental Medicine, Academy of Sciences of the Czech Republic, Prague, Czech Republic; 2 Second Medical Faculty, Charles University, Prague, Czech Republic; 3 Institute for the Study of Nanostructured Material, National Research Council, Bologna, Italy; 4 Laboratory of Gene Expression, Institute of Biotechnology, Academy of Sciences of the Czech Republic, Prague, Czech Republic; 5 Department of Human and General Physiology, University of Bologna, Bologna, Italy; Centre national de la recherche scientifique, University of Bordeaux, France

## Abstract

The polymodal transient receptor potential vanilloid 4 (TRPV4) channel, a member of the TRP channel family, is a calcium-permeable cationic channel that is gated by various stimuli such as cell swelling, low pH and high temperature. Therefore, TRPV4-mediated calcium entry may be involved in neuronal and glia pathophysiology associated with various disorders of the central nervous system, such as ischemia. The TRPV4 channel has been recently found in adult rat cortical and hippocampal astrocytes; however, its role in astrocyte pathophysiology is still not defined. In the present study, we examined the impact of cerebral hypoxia/ischemia (H/I) on the functional expression of astrocytic TRPV4 channels in the adult rat hippocampal CA1 region employing immunohistochemical analyses, the patch-clamp technique and microfluorimetric intracellular calcium imaging on astrocytes in slices as well as on those isolated from sham-operated or ischemic hippocampi. Hypoxia/ischemia was induced by a bilateral 15-minute occlusion of the common carotids combined with hypoxic conditions. Our immunohistochemical analyses revealed that 7 days after H/I, the expression of TRPV4 is markedly enhanced in hippocampal astrocytes of the CA1 region and that the increasing TRPV4 expression coincides with the development of astrogliosis. Additionally, adult hippocampal astrocytes in slices or cultured hippocampal astrocytes respond to the TRPV4 activator 4-alpha-phorbol-12,-13-didecanoate (4αPDD) by an increase in intracellular calcium and the activation of a cationic current, both of which are abolished by the removal of extracellular calcium or exposure to TRP antagonists, such as Ruthenium Red or RN1734. Following hypoxic/ischemic injury, the responses of astrocytes to 4αPDD are significantly augmented. Collectively, we show that TRPV4 channels are involved in ischemia-induced calcium entry in reactive astrocytes and thus, might participate in the pathogenic mechanisms of astroglial reactivity following ischemic insult.

## Introduction

During pathological conditions such as cerebral ischemia, a rapid increase of intracellular calcium ([Ca^2+^]_i_) initiates dramatic changes in the nervous tissue, leading to apoptotic and necrotic cell death and reactive gliosis [1], [2]. There is considerable evidence that the [Ca^2+^]_i_ oscillations and propagating [Ca^2+^]_i_ waves evoked by focal ischemia can spread through the astroglial syncytium for a long distance and cause damage in distal CNS regions [3]. Despite the large number of studies describing the phenomenon of astroglial calcium influx evoked by acute brain injury, data regarding the molecular identity of the ion channels and receptors involved in this event are more elusive. It has been suggested that in astrocytes the massive and uncontrolled plasmalemal Ca^2+^ entry after hypoxia/ischemia could be mediated by the activation of voltage-gated Ca^2+^ channels [4], NMDA receptors [5], P2X7 and P2Y purinergic receptors [6], the reversed operation of the Na/Ca^2+^ exchanger [7] and potentially, Ca^2+^ permeable cation channels such as transient receptor potential (TRP) channels [8]. Previously, it has been shown that in the brain TRP channels are expressed predominantly in neurons. Lipski and co-workers [9] have demonstrated the expression of TRPM2/TRPM7 and TRPV3/TRPV4 in neurons of the CA1 subfield of the hippocampus and suggested their involvement in oxidative stress. Furthermore, Cao and co-authors [10] revealed the co-expression of TRPV1 and TRPV4 in neuronal cell bodies of the dorsal root ganglion (DRG) and found that 4-alpha-phorbol 12, 13-didecanoate (4αPDD) induced an increase of [Ca^2+^]_i_ in DRG neuronal co-cultures. The expression of different TRP channels was also described in glial cells. Numerous investigators have demonstrated the expression of heteromultimeric complexes of TRPC1-, TRPC3-, TRPC4- and TRPC5 channels in embryonic cultured astrocytes and in freshly isolated astrocytes from rat cortices as well as their involvement in the modulation of store-operated Ca^2+^ entry activity [11]–[13].

Of particular interest is a member of the vanilloid subfamily, the TRPV4 channel, which is widely expressed in the brain [14]. TRPV4 channels can be activated by diverse stimuli such as moderate heat, endogenous agonists such as arachidonic acid or the synthetic ligand 4αPDD [15]–[17]. In astrocytes, TRPV4 is also sensitive to hypotonicity, and by forming a molecular complex with aquaporins, it might participate in regulating cell volume recovery [18]–[20]. There is evidence that primary cultured astrocytes as well as cortical astrocytes of the rat neocortex strongly express TRPV4 channels [21]. Typical TRPV4 currents activated by 4αPDD or hypotonicity and blocked by Ca^2+^-free solution or the TRPV4 inhibitor, Ruthenium Red (RR) have been found in cultured astrocytes. A recent study on organotypic slices of the juvenile hippocampus confirmed TRPV4 channel expression in astrocytes and revealed their involvement in oxidative stress-induced cell death [8]. The application of RR or Gd^3+^ reduced astrocytic damage, thus suggesting the involvement of TRPV4 channels in astroglial pathophysiology. However, to the best of our knowledge, the role of astrocytic TRPV4 channels during *in vivo* ischemic injury has not yet been defined.

The present study was undertaken to address the pathophysiological role of TRPV4 channels in adult rat astrocytes. The functional expression of TRPV4 channels in hippocampal astrocytes was investigated after the induction of cerebral hypoxia/ischemia (H/I) by a bilateral occlusion of the carotids combined with hypoxic conditions and followed by reperfusion. By using immunocyto/histochemical and western blot analyses, as well as single cell microfluorimetry and electrophysiological techniques, we have characterized TRPV4 expression in sham-operated rats and those 1 hour after H/I (acute phase of reperfusion) and 7 days after H/I (late phase of reperfusion). We show that TRPV4 expression and activity are up-regulated in astrocytes following ischemia, suggesting that this channel could be involved in the [Ca^2+^]_i_ elevation occurring in the astroglial syncytium as a result of an ischemic insult.

## Materials and Methods

### Ethics Statement

All procedures involving the use of laboratory animals were performed in accordance with the European Communities Council Directive 24 November 1986 (86/609/EEC) and animal care guidelines approved by the Institute of Experimental Medicine, Academy of Sciences of the Czech Republic Animal Care Committee on April 17, 2009; approval number 85/2009.

### Induction of Cerebral Hypoxia/ischemia in Rats

Adult male Wistar rats (200 to 250 g) were pre-medicated with atropine (100 µg/kg, s.c.; Biotika, Slovak Republic) and anesthetized with sodium pentobarbital (PTB, 65 mg/kg, i.p.; Sigma-Aldrich, St. Louis, MO, USA). Hypoxia/ischemia was induced by a bilateral 15-minute occlusion of the common carotids combined with hypoxic conditions as described previously [22], [23]. The rats were intubated by a cannula tube (Abbocath-T 16G, Abbott, Sligo, Ireland) and mechanically ventilated with 33.3% O_2_ and 66.6% N_2_ (rate 60 cycles/min, Linde Gas, Prague, Czech Rep.) using a CIV-101 animal ventilator (Columbus Instruments, Columbus, OH, USA) for 15 minutes prior to carotid artery occlusion and during the first 60 minutes of reperfusion. Body temperature was controlled with a heating pad and maintained at 37±1°C throughout the surgery. Both carotid arteries were exposed and occluded with aneurism clips for 15 minutes. During the occlusion the rats were ventilated with 6% O_2_ and 94% N_2_ (Linde Gas, Prague, Czech Rep.). After 15 minutes of H/I the clamps were removed and the blood flow was renewed. In sham-operated rats, which were used as a control, the common carotid arteries were exposed but not occluded. The rats were left to survive for 1 hour (1H H/I) or 7 days after hypoxia/ischemia (7D H/I). The animals were housed individually and allowed food and water *ad libitum*.

### Acute Brain Slice Preparation for Electrophysiology

After the reperfusion period the rats were deeply anesthetized with a sub-lethal dose of PTB (100 mg/kg, i.p.) and perfused transcardially with cold (4°C, 40 ml) isolation solution containing (in mM): 110 NMDG-Cl, 2.5 KCl, 24.5 NaHCO_3_, 1.25 Na_2_HPO_4_, 0.5 CaCl_2_, 7 MgCl_2_, 20 glucose (pH 7.4, osmolality 290 mOsm/kg). After decapitation, the brains were quickly dissected out and fixed on a cutting disk using 3 M Vetbond tissue adhesive (World Precision Instruments, Sarasota, FL, USA). Then the brains were transferred to a microtome chamber containing cold (4°C) isolation solution gassed with 95% O_2_ and 5% CO_2_. Transversal 220 µm thick slices were cut using an HM 650 V vibration microtome (Thermo Scientific Microm, Walldorf, Germany). Subsequently, the slices were incubated in isolation solution (34°C) for 30 minutes and then kept in artificial cerebrospinal fluid, further termed aCSF (see Table 1), at room temperature for at least 1 hour.

**Table 1:** Composition of the extracellular and intracellular solutions for Ca^2+^ imaging and patch-clamp measurements.

Extracellular solutions used for [Ca^2+^]_i_ measurements *in vitro* and *in situ*									
	NaCl	KCl	MgCl_2_	CaCl_2_	NaHCO_3_	Na_2_HPO_4_	Glucose	mOsm/kg	
**aCSF**	122	3	1.3	1.5	28	1.25	10	305±5	
**aCSF_ØCa_**	122	3	1.3	–	28	1.25	10	305±5	
**Extracellular solutions used for whole-cell patch-clamp recordings ** ***in vitro*** ** and ** ***in situ***									
	**NaCl**	**KCl**	**MgCl**	**CaCl_2_**	**HEPES**	**CsCl**	**Glucose**	**mOsm/kg**	**pH**
**Ext1**	140	4	2	2	10	–	5	315±5	NaOH
**Ext2**	–	–	2	10	10	122	5	315±5	CsOH
**Ext2_ØCa_**	–	–	2	–	10	122	5	315±5	CsOH
**Intracellular solutions used for whole-cell patch-clamp recordings ** ***in vitro*** ** and ** ***in situ***									
	**CsGluc**	**CsCl**	**KCl**	**CaCl_2_**	**MgCl_2_**	**EGTA**	**HEPES**	**mOsm/kg**	**pH**
**Int1**	–	–	130	0.5	2	5	10	280±5	KOH
**Int2**	100	26	–	–	2	1	10	300±5	CsOH

Abbreviations: extracellular solution (Ext); intracellular solution (Int). All concentrations are in mM. The pH of the extracellular solutions was adjusted to 7.4 with NaOH in Ext1 or with CsOH in Ext2 and Ext2_ØCa_, while the pH of the intracellular solutions was adjusted to 7.2 with KOH in Int1 or CsOH in Int2_._ Osmolality was adjusted with mannitol in Ext1, Ext2, Ext2_ØCa_ and Int1 solutions.

### Preparation of Primary Cultures of Dissociated Astrocytes from the CA1 Region of the Hippocampus

Primary cultured astrocytes were prepared from all experimental groups of rats: sham-operated rats and those 1 hour and 7 days after hypoxia/ischemia. Transcardial perfusion and slice preparation were performed as described above, while the thickness of the coronal slices was 700 µm. The hippocampal CA1 region was dissected out from the slice, cut into small pieces and transferred into a Falcon tube with 4 ml of Dubelcco’s Modified Eagle Medium (DMEM, Gibco-Invitrogen, Carlsbad, CA, USA) containing 15% fetal bovine serum (FBS, PAA Laboratories GmbH, Pasching, Austria), then centrifuged for 3 minutes at 2000 rpm. The supernatant was discarded and the tissue was dissociated by a micropipette in 2 ml of solution containing 0.05% Trypsin and 2 g/l ethylene-diamine-tetraacetic acid (EDTA, Sigma-Aldrich, St. Louis, MO, USA). After 3 minutes, the dissociated cells were transferred into Falcon tubes containing 2 ml of FBS solution to block the trypsinization. After centrifugation (3 min at 2000 rpm), the supernatant was removed and fresh cultivation medium (DMEM containing 15% FBS) was added up to 6 ml. The cells were resuspended and plated in 0.5 ml volumes onto 12 poly-L-lysine (PLL, Sigma-Aldrich, St. Louis, MO, USA)-coated coverslips. Cells were cultured in DMEM containing 15% FBS in an incubator (100% humidity, 5% CO_2_) at 37°C with medium exchange on the fourth day. After 4–5 days the cells were used for immunocytochemistry, patch-clamp and [Ca^2+^]_i_ measurements.

### Solutions and Reagents

#### Solutions

Various extracellular and intracellular solutions were used in our experiments and they are listed in Table 1.

For microfluorimetric analyses of intracellular calcium ([Ca^2+^]_i_) *in vitro* and *in situ*, the extracellular solutions aCSF and aCSF_ØCa_ were used. For the electrophysiological characterization of astrocytes *in situ* and *in vitro*, the extracellular solution aCSF and the intracellular solution Int1 were used, whereas recordings for TRPV4 channel characterization were obtained using the extracellular solution Ext1 and the intracellular solution Int2 as previously described by Benfenati and co-authors [21]. In order to facilitate the isolation of TRPV4 currents, Na^+^ and K^+^ conductances were eliminated by replacing Na^+^ and K^+^ with Cs^+^ in the extracellular (Ext2) and intracellular solutions (Int2). In the intracellular solution, Cl^-^ was partially replaced by gluconate to diminish the activation of astroglial Cl^-^ conductance [24]. All chemicals were purchased from Sigma-Aldrich, St. Louis, MO, USA.

#### Agonists and antagonists

The TRPV4 activator 4-alpha-phorbol 12, 13-didecanoate (4αPDD, Sigma-Aldrich, St. Louis, MO, USA) was applied at 5 µM for *in vitro* and 5–10 µM for *in situ* studies. Cells were exposed to 4αPDD for 6–8 min. 4αPDD was kept in aliquots at −20°C in dimethyl sulfoxide (DMSO, Sigma-Aldrich, St. Louis, MO, USA). Aliquots of Ruthenium Red (RR, Sigma-Aldrich, St. Louis, MO, USA) and RN1734 (Tocris Bioscience, Bristol, UK) were also prepared in DMSO. Aliquots were stored at −20°C, and the solutions were prepared immediately before use (the final concentration of DMSO was less than 0.03%).

### Patch-clamp Recording

Membrane currents were recorded using the patch-clamp technique in the whole-cell configuration in slices as well as in primary cultured astrocytes. Recording pipettes with a tip resistance of 8–12 MΩ were made from borosilicate capillaries (0.86 ID, Sutter Instruments Company, Novato, CA, USA) using a P-97 Brown-Flaming micropipette puller (Sutter Instruments, Novato, CA, USA). To visualize the recorded cells, the intracellular solution contained either Lucifer Yellow (LY, Sigma-Aldrich, St. Louis, MO, USA) or Alexa-Fluor hydrazide 488/594 (Molecular Probes, Carlsbad, CA, USA). The LY-labeled cells were used for further post-recording immunocytochemical identification. Since TRPV4 is a moderately heat sensitive channel [16], the electrophysiological experiments were performed at 28±2°C. Electrophysiological data were measured with 10 kHz sample frequency using an EPC10 amplifier controlled by TIDA or PatchMaster software (HEKA Elektronik, Lambrecht/Pfalz, Germany) and were filtered at 2.9 kHz using a Bessel filter. Acute brain slices or coverslips with primary astrocyte cultures were transferred to the recording chamber of an upright Axioscop microscope (Zeiss, Gottingen, Germany) equipped with electronic micromanipulators (Luigs &Neumann, Ratingen, Germany) and a high-resolution AxioCam HRc digital camera (Zeiss, Germany).

Resting membrane potential (V_rest_) and membrane capacitance (C_m_) were measured as described previously [25]. Input resistance (IR) was determined from the currents evoked by membrane depolarization from −70 mV to −60 mV, 40 ms after the onset of depolarization. Electrophysiological data were analyzed using TIDA and Fitmaster software (HEKA, Lambrecht, Germany). Recorded membrane potentials were corrected for the liquid junction potential using JPCALCW software [26].

### Microfluorimetric Analysis of Intracellular Calcium Levels

Coverslips with 4–5-day-old cell cultures were incubated for 1 hour in 1.5 ml DMEM +15% FBS medium containing 3.3 µM Fluo-4 AM and 0.07% Pluronic F-127 (Invitrogen, Carlsbad, CA, USA) at 37°C in an incubator (100% humidity, 5% CO_2_). The coverslips were then transferred to a microscope superfusion chamber, and two measurements were made on each coverslip with a sufficient distance between the measurement regions. After the measurements, immunocytochemical staining for GFAP was performed to confirm the astrocytic identity of the measured cells.

*In situ* measurements were performed in the *stratum radiatum* of the CA1 region of the hippocampus, 10–20 µm below the surface of the slice. As already described, 220 µm thick transversal brain slices were cut in 4°C isolation solution using a vibration microtome. They were then incubated for 20 min in isolation solution gassed with 95% O_2_ and 5% CO_2_ containing 1 µM Sulforhodamine 101 (SR-101, Sigma-Aldrich, St.Louis, MO, USA) at 34°C. The slices were then placed onto a nylon mesh in a dish (3 ml volume) and incubated for 60 min in aCSF containing 4 µM Fluo-4 AM and 0.01% Pluronic F-127 at room temperature. During incubation, the dish was in a dark environment and gassed with 95% O_2_ and 5% CO_2_. The slices were then mounted on a microscope superfusion chamber, and two measurements were performed with a sufficient distance between them. For verification that the measured cells were astrocytes, SR-101 staining was used since this fluorescent dye is preferentially taken up by astrocytes [27].

During the measurements, the microscope superfusion chamber was continually perfused with aCSF at a flow rate of 2.5 ml/min. The temperature was held at 28±2°C throughout the experiments by a ThermoClamp-1 (AutoMate Scientific, Inc. Berkeley, CA, USA). The solutions were applied through a capillary (i.d. 250 µm) located 0.5–1 mm from the measurement region and connected to a Perfusion Pressure Kit pressurized application system (flow rate 600 µl/min) controlled by a ValveBank II controller (AutoMate Scientific, Inc. Berkeley, CA, USA). Since TRPV4 channels are stretch-sensitive [28], aCSF was applied before and after 4αPDD application with the same flow rate to verify that the response was not influenced by the application itself. Fluo-4 fluorescence was detected with a TILL Photonics Imaging System installed on a Zeiss Axioskop 2 FS Plus microscope equipped with a long-distance 10× objective (Achroplan 0.3 W, Ph 1, Zeiss, Germany) for measurements *in vitro* or a long-distance 40× objective (IR Achroplan 0.8 W, Zeiss, Germany) for measurements *in situ*. A digital camera (PCO Sensicam, Kelheim, Germany) was controlled by TILLvisION software. The excitation light (488 nm) was generated by a Polychrome V (TILL Photonics GmbH, Gräfelfing, Germany), filtered by a BP 450–490 excitation band-pass filter, reflected by a FT 510 beam splitter and the emitted light was filtered by a LP 515 long-pass filter (Filter Set 09, Zeiss, Germany). Images were acquired at 0.83 Hz and were analyzed offline. Fluorescence intensity (F) was measured in the cell bodies and expressed as (F−F_0_)/F_0_ after background correction, where F_0_ is the baseline fluorescence intensity before drug application. The threshold for a Ca^2+^ response *in vitro* was 120% of F_0_, and the threshold amplitude for a transient peak *in situ* was 200% of the amplitude of the noise signal. The excitation light for SR-101 (570 nm) was filtered by a BP565/30 band-pass filter, reflected by a FT585 beam splitter and the emitted light was filtered by a 620/60 band-pass filter (Filter Set 31, Zeiss, Germany).

### Immunocytochemistry/Immunohistochemistry

*Primary cultured astrocytes* attached to PLL-coated coverslips were fixed on the 4–5th day of culture in 4% paraformaldehyde solution in 0.2 M phosphate buffer (PB, pH 7.4) for 8 minutes and kept in 10 mM phosphate buffered saline (PBS) at 4°C for further processing. Coverslips were incubated in a blocking solution containing 5% Chemiblocker (Millipore, Billerica, MA, USA) and 0.5% Triton X-100 (Sigma-Aldrich, St. Louis, MO, USA) in 10 mM PBS at 4°C for two hours. They were then incubated overnight at 4°C with a rabbit anti-TRPV4 antibody (1∶200; Novus Biologicals, Littleton, CO, USA) in PBS containing 0.2% Triton X-100. After overnight incubation, three 10-min washes with PBS were performed, followed by incubation with Alexa 488-conjugated goat anti-rabbit IgG- (1∶200; GAR-488, Molecular Probes, Carlsbad, CA, USA) for two hours at 4°C. For double labeling, a mouse monoclonal antibody directed against GFAP and conjugated with Cy3 (1∶800; Sigma-Aldrich, St. Louis, MO, USA) was subsequently applied and incubated overnight at 4°C. Afterwards, the coverslips were washed in PBS three times for 10 minutes each. To visualize the cell nuclei, the coverslips were incubated with 300 nM 4′,6-diamidino-2-phenylindole (DAPI) in PBS for 5 minutes at room temperature. Finally, the coverslips were mounted using Aqua Poly/Mount (Polysciences Inc., Eppelheim, Germany).

*Brain slices* were prepared from control rats and those 1H and 7D after H/I. Animals were anesthetized with a sub-lethal dose of PTB (100 mg/kg, i.p.) and perfused transcardially with 70 ml of 0.9% saline with heparin (2500 IU/100 ml), (Zentiva, Prague, Czech Republic), followed by 70 ml of 4% paraformaldehyde solution in PBS (PFA/PBS). Dissected brains were postfixed overnight in 4% PFA/PBS, cryoprotected for several hours in a series of 10%, 20% and 30% sucrose in 0.2 M PB, then sectioned in the coronal plane (30 µm thickness). To enhance the efficiency of immunostaining the sections were incubated for 20 minutes in citrate buffer (10 mM Citric Acid, 0.05% Tween 20 in PB, pH 6.0) at 80°C. After washout in PB, the sections were further incubated in a blocking solution containing 2% normal goat serum (NGS, Millipore, Billerica, MA, USA), 5% Chemiblocker, 1% bovine serum albumin (BSA, Sigma-Aldrich, St. Louis, MO, USA) and 0.5% Triton X-100 in PB for 2 hours at 4°C. An epitope-specific rabbit anti-TRPV4 (1∶500) and Cy3-conjugated mouse anti-GFAP (1∶800) were diluted in 5% Chemiblocker and incubated with the sections overnight at 4°C. After incubation, three 10-min washes with PBS removed the unbound antibodies. Alexa 488-conjugated goat anti-rabbit IgG (1∶200) (GAR-488, Invitrogen/Molecular Probes, Carlsbad, CA, USA) was applied and the slices were incubated for two hours at 4°C. Slices were then washed in PBS and mounted with Vectashield containing DAPI (Vector Laboratories, Burlingame, CA) for visualization of the nuclei. For immunohistochemical analysis of the hippocampus after hypoxia/ischemia, mouse anti-NeuN (1∶100; Millipore, Billerica, MA, USA) and Cy3-conjugated mouse anti-GFAP (1∶800) primary antibodies were used. For cell identification after patch-clamp recording, the measured cells were filled either with Alexa Fluor 488 hydrazide or LY by dialyzing the cytoplasm with the patch pipette solution. Post-recording, the coverslips and slices were fixed with 4% PFA in 100 mM PB for 8 min or 1 hour, respectively. After incubation in a blocking solution containing 5% Chemiblocker and 0.5% Triton X-100 in 10 mM PBS, Cy3-conjugated mouse anti-GFAP (1∶800) was applied for 2 hours at 4°C. The specificity of the TRPV4 staining was confirmed by strong TRPV4 immunostaining in the choroid plexus, as described previously [8], [16]. The slices/coverslips were then examined using an LSM 5 DUO spectral confocal microscope (Zeiss, Germany).

### Western Blotting

Hippocampal samples for Western blot analysis were isolated from rats after H/I (1H, 6H, 1D, 3D, 7D), sham-operated animals (6H, 3D) or intact animals. Rats were deeply anesthetized with PTB (100 mg/kg, i.p.), perfused transcardially with chilled isolation solution and decapitated. The brains were dissected out and cut into 500 µm thick slices. The CA1 region of the hippocampus was excised from each slice and homogenized using an ultrasound homogenizator in Tris buffer (pH 6.8) containing 10% glycerol and 1% sodium dodecyl sulphate (SDS). Total protein content in the homogenates was determined by the Micro BCA™ protein assay kit (ThermoFisherScientific, Rockford, IL, USA). Tissue homogenates were heated at 100°C for 5 minutes with 0.5% dithiothreitol. Equal amounts of proteins were separated on a 6% SDS-polyacrylamide gel (for TRPV4 protein) or 10% SDS-polyacrylamide gel (for GFAP and β-actin) and subsequently electrotransferred to a nitrocellulose membrane using a TE 70XP Semi-Dry Transfer unit (Hoefer, Holliston, MA, USA). Membranes were blocked with 5% non-fat dry milk in PBS–Tween buffer (0.05% Tween) for 1 hour at room temperature. The incubation with the primary antibodies diluted in PBS containing 1% non-fat dry milk, 0.05% Tween and 0.1% NaN_3_ was performed at 4°C overnight, followed by a 2 hour incubation with goat anti-rabbit IgG conjugated with peroxidase (Sigma–Aldrich, St. Louis, MO, USA) at room temperature. The following primary antibodies were used: rabbit anti-GFAP (1∶600, Sigma–Aldrich, St. Louis, MO, USA), rabbit anti-TRPV4 (1∶300; Alomone Labs, Jerusalem, Israel) and rabbit anti-β-actin (Abcam, Cambridge, UK). SuperSignal West Pico Chemiluminiscent Substrate (Thermo Fisher Scientific, Rockford, IL, USA) was used to develop the Western blots. To quantify the changes in TRPV4 protein content, Quantity One software (Bio-Rad Laboratories, Hercules, CA, USA) was employed for imaging and analyzing 1-D electrophoresis gels. Quantification was carried out using 3 independent Western blot analyses (**Fig. S1**).

### Quantitative PCR Analyses

The hippocampal CA1 regions from 3 sham-operated and 3 ischemic rats (1H after H/I) were immediately placed into RLT buffer after dissection (Qiagen, Gemany). Total RNA was extracted using an RNeasy Mini Kit, including DNase treatment (Qiagen, Germany). RNA concentrations were measured with a NanoDrop ND-1000 spectrophotometer (Nanodrop Technologies). Using SuperScript III (Life Technologies), reverse transcription was performed as described recently [29]. Real-time PCR measurements were performed using a Biorad CFX (Bio-Rad Laboratories, Hercules, CA, USA) and a temperature profile of 95°C for 3 minutes, followed by 40 cycles at 95°C for 15 s, 60°C for 15 s, and 72°C for 20 s. The ten-microliter reactions contained iQ SYBR Green Supermix (Bio-Rad Laboratories, Hercules, CA, USA) and 400 nM of each primer (Metabion, Germany). The employed primer sequences were: *Trpv4*_forward: TTTGCTCTTATTTCTACTCCTCCC and *Trpv4*_reverse: GCTGGCTTAGGTGACTCC. Reference genes were evaluated using the Mouse Endogenous Control Gene Panel (TATAA Biocenter, Sweden) and NormFinder. All data were normalized against β2-microtubulin (*B2M)*. Relative changes in expression were calculated using the ΔΔCq Method [30].

### Data Analysis and Statistics

Data are presented as means ± S.E.M. (standard error of the mean) for *n* cells. Student’s unpaired *t*-test or one-way ANOVA for multiple comparisons were used to determine significant differences between the experimental groups. Values of *p<0.05 were considered significant, **p<0.01 very significant and *** p<0.001 extremely significant.

## Results

### Increased TRPV4 Immunoreactivity in Hippocampal Astrocytes Coincides with the Development of Astrogliosis

**Figure 1:**
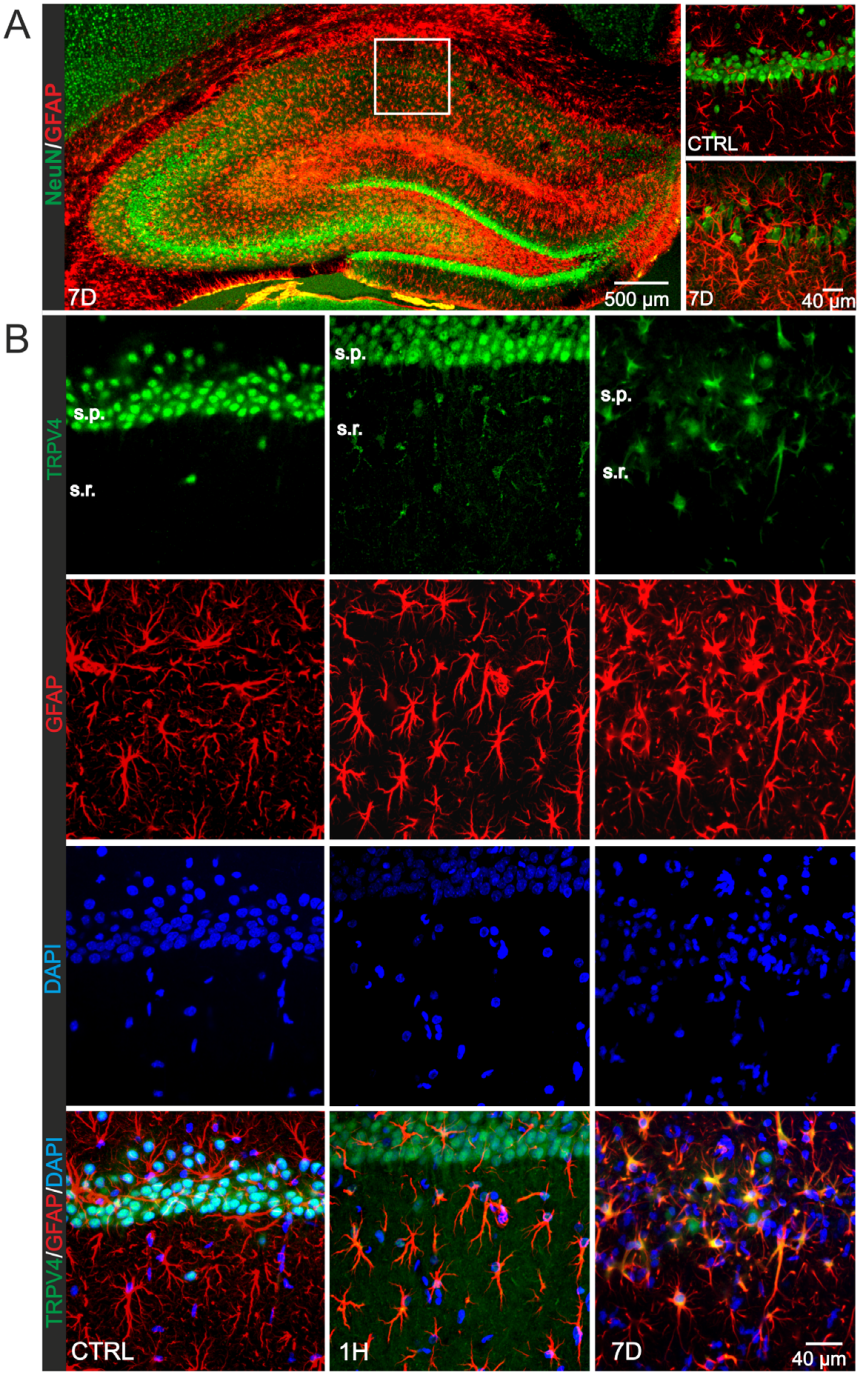
Immunohistochemical analyses of the rat hippocampus after hypoxia/ischemia followed by reperfusion. (**A**) Coronal sections of the rat hippocampus immunostained for a neuronal marker (NeuN) and the astrocytic marker glial fibrillary acidic protein (GFAP) in sham-operated rats (CTRL) and those 7 days (7D) after hypoxia/ischemia (H/I). Enlargements of the tissue section shown on the right demonstrate pyramidal cell loss and the formation of reactive gliosis in the hippocampal CA1 region 7D after H/I when compared to controls. (**B**) TRPV4 immunostaining in the CA1 region of the hippocampus. Coronal slices from controls and ischemic rats were labeled for TRPV4 (green) and GFAP (red). Note that in controls the TRPV4 immunoreactivity was detected in pyramidal cells and more rarely in astrocytes. With developing astrogliosis TRPV4 immunoreactivity was increased in astrocytes. Seven days after H/I no TRPV4 expression was detected in pyramidal cells, whereas it was markedly increased in astrocytes. The following abbreviations are used: H/I (hypoxia/ischemia), CTRL (sham-operated rats), 1H (1 hour), 7D (7 days) after hypoxia/ischemia, s.p. (stratum pyramidale), s.r. (stratum radiatum).

**Figure 2:**
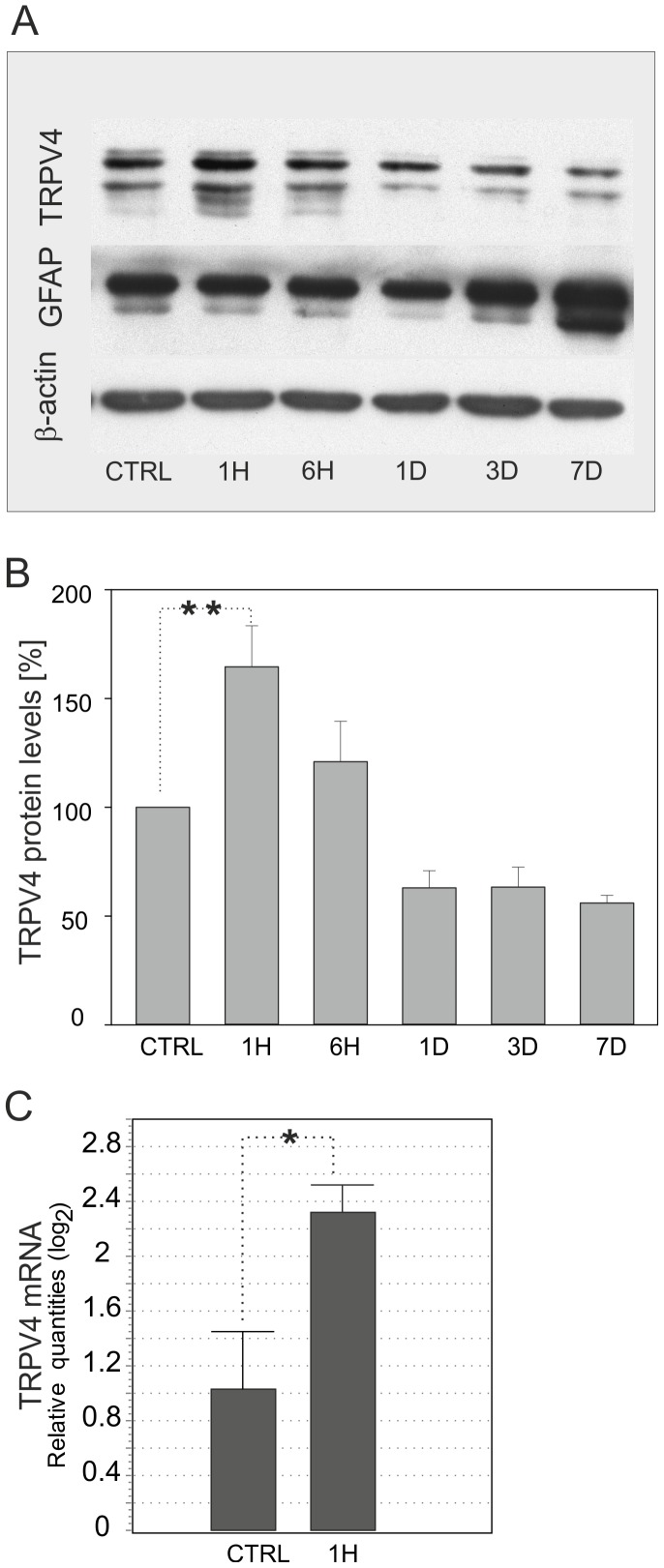
Western blot and PCR analyses of TRPV4 protein in the CA1 region of the hippocampus after hypoxia/ischemia. (**A**) Time-dependent changes in the expression of TRPV4, GFAP and β-actin proteins in the CA1 region of the hippocampus of sham-operated rats (CTRL), 1 hour (1H), 6 hours (6H), 1 day (1D), 3 days (3D) and 7 days (7D) after hypoxia/ischemia. Note that the expression of GFAP gradually increased 3 and 7 days after ischemia. β-actin was used as a loading control. (**B**) Time-dependent changes in TRPV4 protein levels showing the significant increase in TRPV4 protein 1H after H/I. Data were obtained from 3 independent Western blot analyses. (**C**) Quantitative RT-PCR revealing significantly increased TRPV4 mRNA levels 1H after H/I compared to sham-operated rats. Data were obtained from 3 independent isolations of total mRNA from the hippocampal CA1 region. Statistical significance was calculated using one-way ANOVA and Dunnett’s multiple comparison test; *p<0.05 significant, **p<0.01 very significant.

To investigate the expression of TRPV4 channels in astrocytes and their possible contribution to hypoxia/ischemia-induced astroglial reactivity, we performed immunohistochemical analyses of the rat hippocampus in control and ischemic rats **(** **Fig. 1A, B** **)**. As previously described by Anderova and co-authors [22], hypoxic/ischemic injury was characterized by a decrease in NeuN immunoreactivity due to neuronal loss occurring within 3–7 days after hypoxia/ischemia (H/I) and by an increase in GFAP immunoreactivity due to the development of astrogliosis within 7D after H/I (**Fig. 1A**). In control hippocampal sections double immunostaining with antibodies against TRPV4 and GFAP revealed neuronal TRPV4 immunoreactivity in the *stratum pyramidale* of the CA1 region and only a few TRPV4-positive astrocytes in the *stratum radiatum* of controls **(** **Fig. 1B** **)**. However, TRPV4 immunoreactivity gradually increased in astrocytes with the progression of reactive gliosis in response to H/I, and it declined in CA1 pyramidal neurons in conjunction with ongoing neuronal cell death. Within 7 days of reperfusion we have observed a shift in TRPV4 immunoreactivity from neurons to reactive astrocytes (data not shown)**,** leading to TRPV4 expression only in reactive astrocytes **(** **Fig. 1B** **)**. No pyramidal cells positive for TRPV4 were found in the CA1 hippocampal region after 7 days of reperfusion. To verify the molecular expression of TRPV4 after H/I, we performed Western blot analyses of protein lysates extracted from the CA1 region of the hippocampus in control rats and those 1 hour (1H), 6 hours (6H), 1 day (1D), 3 days (3D) and 7 days (7D) after H/I. TRPV4-positive bands of molecular weight ∼120 kDa were detected with an increase in TRPV4 protein levels 1H after H/I, while 1D and 7D after H/I the overall expression of TRPV4 was down-regulated **(** **Fig. 2** **and Fig. S1)**. Additionally, quantitative real-time PCR analyses revealed that there was a 2.4-fold increase in TRPV4 mRNA 1 hour after H/I when compared to the mRNA content of the hippocampal CA1 region in sham-operated rats **(** **Fig. 2** **).** As expected for an increase in astroglial reactivity, GFAP was gradually increased 3D and 7D after ischemia **(** **Fig. 2** **and Fig. S1)**. The apparent discrepancy in TRPV4 expression between immunocytochemistry and Western blotting could be due to the fact that following H/I, neuronal viability decreases, and this could account for the overall decrease in TRPV4 protein levels observed in total cell lysates of the hippocampal CA1 region.

### TRPV4-mediated Responses in Reactive Astrocytes are Augmented *in Situ* after Hypoxia/ischemia

**Figure 3:**
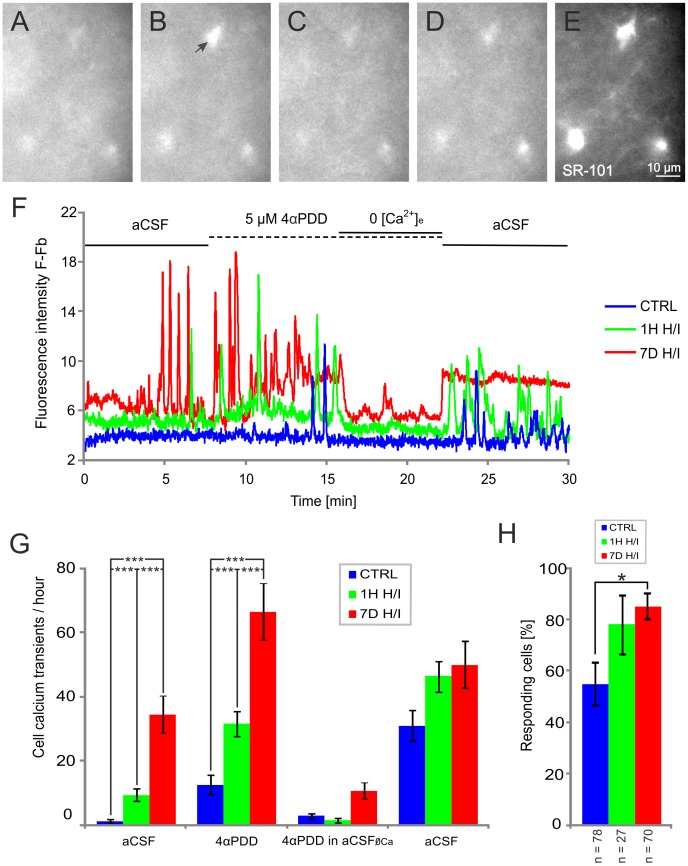
The TRPV4 agonist 4αPDD triggers Ca^2+^ oscillations in astrocytes of the hippocampal CA1 region. (**A–D**) Astrocytes in acute rat hippocampal slices loaded with the calcium fluorescent probe Fluo-4 AM respond with an increase in fluorescence to the application of the TRPV4 channel agonist 4αPDD (5 µM). (A) Cells before 4αPDD application in aCSF, (**B**) during 4αPDD application, (**C**) during 4αPDD application in aCSF without Ca^2+^ (aCSF_ØCa_) and (**D**) during washout with aCSF. Note the increased fluorescence indicated by the arrow in B. (**E**) Cells loaded with Sulforhodamine 101 (SR-101) to verify that the measured cells are astrocytes. (**F**) Representative fluorescence traces with background correction of astrocytes in acute slices prepared from sham-operated rats (CTRL) and rats 1 hour (1H H/I) and 7 days (7D H/I) after hypoxia/ischemia. (**G**) Histogram of the mean intracellular calcium transients per hour before 4αPDD application (aCSF), during 4αPDD application (4αPDD), during 4αPDD application in aCSF_ØCa_ (4αPDD in aCSF_ØCa_) and following washout (aCSF), measured in astrocytes from acute hippocampal slices prepared from the brains of sham-operated rats (CRTL) and those 1 hour (1H H/I) and 7 days (7D H/I) after hypoxia/ischemia. (**H**) Histogram of the number of responding cells (n =  number of all analyzed cells). The values are presented as mean ± S.E.M. Statistical significance was calculated using one-way ANOVA. *p<0.05, significant; ***p<0.001, extremely significant.

**Figure 4:**
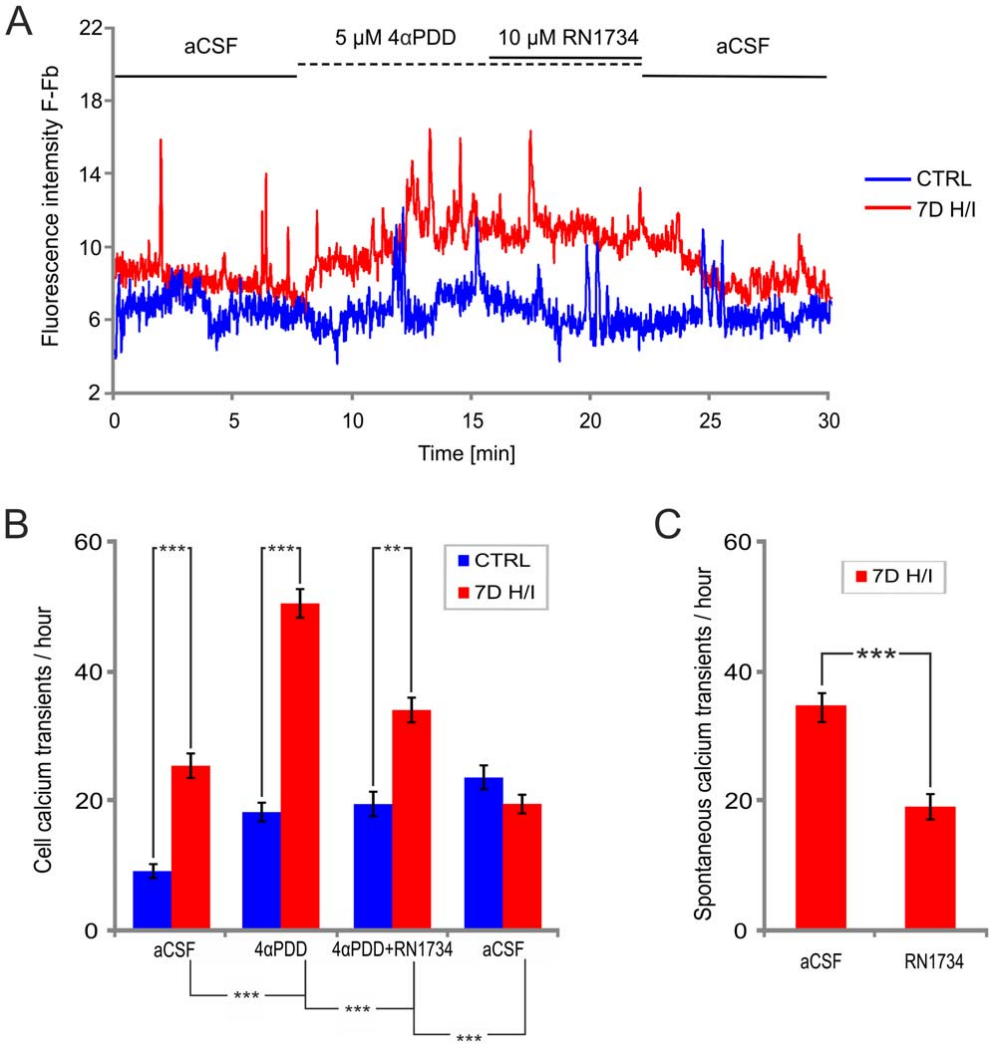
The TRPV4 antagonist RN1734 decreases 4αPDD-induced and spontaneous Ca^2+^ oscillations in astrocytes of the hippocampal CA1 region 7 days after ischemia. (**A**) Representative fluorescence traces of hippocampal astrocytes in slices prepared from sham-operated rats (CTRL) and rats 7 days after hypoxia/ischemia (7D H/I) before 4αPDD application (aCSF), during 5 µM 4αPDD application, during the application of 5 µM 4αPDD with 10 µM RN1734 and following washout (aCSF). (**B**) Histogram of the mean intracellular calcium transients per hour before 4αPDD application (aCSF), during the application of 5 µM 4αPDD, during the application of 5 µM 4αPDD +10 µM RN1734 and following washout in aCSF, in astrocytes in hippocampal slices prepared from the brains of sham-operated rats (CRTL, n = 43) and those 7 days after hypoxia/ischemia (7D H/I, n = 40). (**C**) Histogram of the mean spontaneous intracellular calcium transients per hour before RN1734 application (aCSF) and during the application of 10 µM RN1734 (RN1734), measured in astrocytes from acute hippocampal slices 7 days after hypoxia/ischemia (7D H/I, n = 10). The values are presented as mean ± S.E.M. Statistical significance was calculated using one-way ANOVA in (B) and a paired t-test in (C); ***p<0.001 extremely significant, **p<0.01 very significant.

Having demonstrated that TRPV4 expression in astrocytes increases in response to H/I, we next sought to elucidate the impact of ischemic injury on TRPV4 channel activity in astrocytes in the CA1 region of the hippocampus by analyzing the changes in [Ca^2+^]_i_ signals. Typical images of microfluorimetric [Ca^2+^]_i_ measurements of astrocytes *in situ* are shown in **Figure 3 A-D.** Spontaneous transient [Ca^2+^]_i_ spikes measured in aCSF were typically observed in astrocytes from non-ischemic rats **(** **Fig. 3F**, blue trace, CTRL, n = 35). The oscillation frequency of these spontaneous transient Ca^2+^ spikes significantly increased with the time of reperfusion **(** **Fig. 3F**, 1H after H/I - green trace, n = 27; 7D after H/I - red trace, n = 30), with a ∼20-fold increase in astrocytes 7D after H/I when compared to controls **(** **Fig. 3G** **)**. The subsequent application of 5 µM 4αPDD, a selective TRPV4 agonist [31], resulted in an additional increase in spike frequency **(** **Fig. 3G** **)**. In a few astrocytes 1H after H/I and in most of the astrocytes 7D after H/I, 4αPDD evoked an increase in the amplitude of the [Ca^2+^]_i_ signals **(** **Fig. 3F** **).** Although initially 4αPDD application evoked [Ca^2+^]_i_ oscillations similar to those observed in astrocytes of controls or those 1H after H/I, in astrocytes 7D after H/I prolonged exposure to 4αPDD resulted in the generation of sustained Ca^2+^ entry. The application of 4αPDD in the absence of extracellular Ca^2+^ (aCSF_ØCa_) immediately and almost completely abolished the Ca^2+^ spike activity, which was restored upon re-addition of Ca^2+^. During washout, in some of the astrocytes 7D after H/I, the elevated signal amplitude was restored, but distinct spikes were no longer detectable. The application of 5 µM 4αPDD in the presence of 10 µM RN1734, a novel TRPV4 inhibitor [32], abolished ∼70% of the Ca^2+^ spike activity in astrocytes 7D after H/I, which was not restored after washout in aCSF **(** **Fig. 4** **)**. We also found that 7D after H/I the spontaneous Ca^2+^ transients are partly mediated by TRPV4 channels as the application of RN1734 blocked ∼45% of such spontaneous Ca^2+^ transients**(** **Fig. 4C** **)**. Interestingly, 7 days after H/I 4αPDD not only increased spontaneous Ca^2+^ transients, but also resulted in sustained Ca^2+^ entry as demonstrated in **Fig. 3F** (red line), represented by an increase in the baseline fluorescence intensity. Such sustained Ca^2+^ entry, which was always completely blocked in the absence of extracellular Ca^2+^, was rare in controls (1cell out of 35); however, it increased with the time of reperfusion: it was detected in 4 cells out of 27 1H after H/I and in17 cells out of 30 7D after H/I. Surprisingly, RN1734 blocked only Ca^2+^ transients; we never observed a decline of sustained Ca^2+^ entry in response to this inhibitor **(** **Fig. 4A**, red line**)**. The incidence of 4αPDD-specific responses increased in post-ischemic astrocytes; only 56% of astrocytes from controls exhibited a 4αPDD-evoked increase in [Ca^2+^]_i_, oscillations, while 78% and 84% of astrocytes responded to 4αPDD application 1 hour and 7 days after H/I, respectively. These results indicate that the increased expression/immunoreactivity of TRPV4 in ischemic astrocytes is accompanied by enhanced TRPV4-mediated Ca^2+^ oscillations.

**Figure 5:**
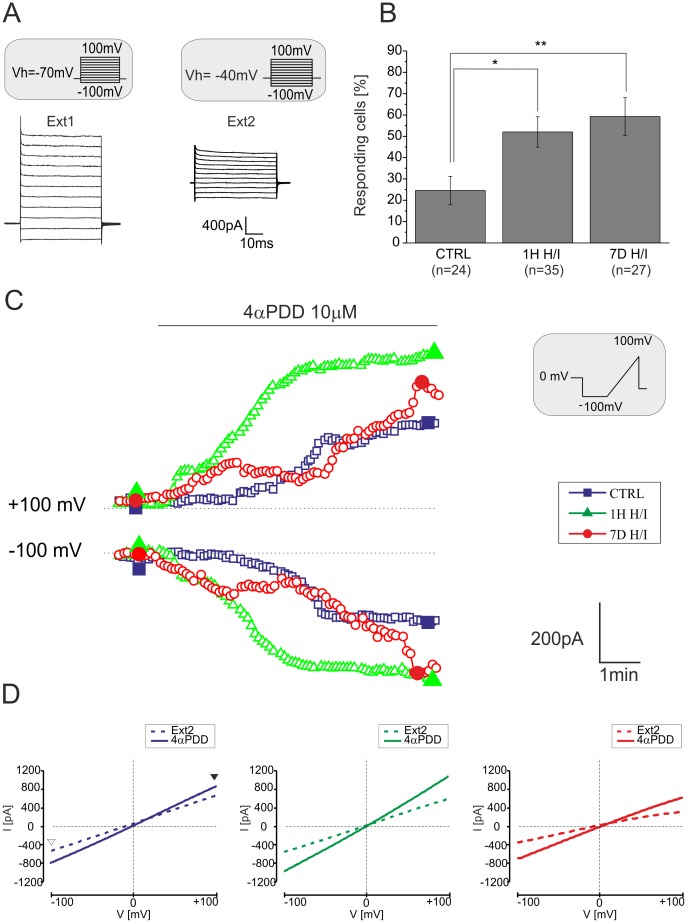
4αPDD-evokes an increase in membrane conductance in astrocytes *in situ.* (**A**) Typical current pattern of astrocytes *in situ* recorded in the CA1 region of the hippocampus 7 days after H/I at a holding potential of −70 mV (**left,** the extracellular solution Ext1 contained K^+^ and Na^+^) and at −40mV (**right,** K^+^ and Na^+^ were replaced by Cs^+^ in the extracellular solution Ext2). (**B**) Percentage of hippocampal astrocytes from sham-operated rats (CTRL), and rats 1H and 7D after H/I that responded to 10 µM 4αPDD (n =  number of cells). The threshold for a 4αPDD current response was 120% of the control ramp current (prior to agonist application), at a voltage of +/−100 mV. The values are presented as mean ± S.E.M. Statistical significance was calculated using one-way ANOVA. *p<0.05, significant; **p<0.01, very significant. (**C**) Time course of 4αPDD-evoked currents measured from the ramp protocol in astrocytes of controls (blue squares) and astrocytes 1H (green triangles) and 7D after H/I (red circles). Currents were measured at −100 mV (white arrowhead) and +100 mV (black arrowhead) in response to a voltage ramp stimulation protocol (see the inset). (**D**) Representative traces of steady state currents (same cells as in C) recorded before (in Ext2 solution, dashed line) and during 4αPDD application (full line) in hippocampal astrocytes of controls (left) and those 1H (middle) and 7D after H/I (right). Representative traces of steady state currents were obtained at the times indicated by the filled blue squares, green triangles and red circles in C. White and black arrowheads indicate the applied voltage ramp and the corresponding current traces (see the inset in C).

**Figure 6:**
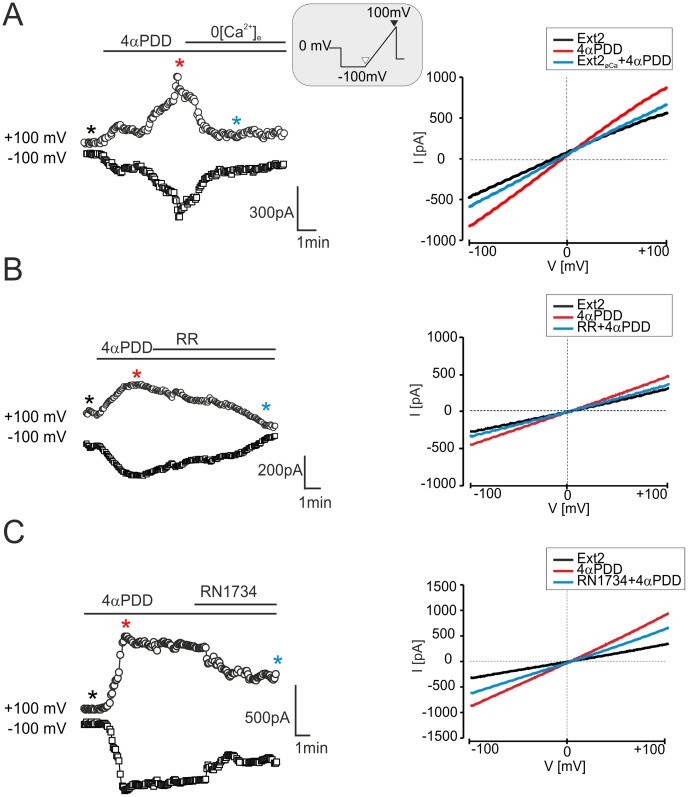
Currents evoked by 4αPDD in hippocampal astrocytes *in situ* are reduced by calcium-free extracellular solution, Ruthenium Red or RN1734. (**A–C, left**) Time course of 4αPDD-evoked currents measured from the ramp protocol in astrocytes 7D after H/I (for the voltage protocol see the inset) prior to and during 4αPDD (10 µM) application and after removing extracellular Ca^2+^ (Ext2_ØCa_, **A**) or after the application of TRPV4 inhibitors, such as Ruthenium Red (RR, 10 µM, **B**) or RN1734 (10 µM, **C**). (**A-C, right**) The traces of the steady state currents (same cells as in left) obtained in Ext2 solution (black lines), during 4αPDD application (red lines) and after removing extracellular Ca^2+^ (Ext2_ØCa_, **A**) or after the application of TRPV4 inhibitors, such as Ruthenium Red (RR, 10 µM, **B**) or RN1734 (10 µM, **C**), are indicated by blue lines. Representative traces of steady state currents were obtained at the times indicated by asterisks of the corresponding colors.

Next, we examined the effect of H/I on TRPV4-mediated currents in astrocytes *in situ*. The typical membrane current patterns of hippocampal astrocytes in control and ischemic tissue were identified in Ext1 by hyper- and depolarizing the astrocytic membrane from −100 mV to +100 mV in 20 mV increments, with the holding potential (V_h_) at −70 mV **(** **Fig. 5A** **)**. Generally, the mature hippocampal astrocytes displayed a passive current pattern, characterized by time- and voltage-independent currents, mainly carried by K^+^ channels, and their passive membrane properties are listed in **Table 2**. In agreement with our previously published data [23], the CA1 hippocampal astrocytes were slightly depolarized 7 days after H/I. To isolate more accurately the TRPV4-specific cationic currents, further recordings were performed in intra- and extracellular solutions in which K^+^ and Na^+^ were replaced with Cs^+^ [21]. Under these experimental conditions, membrane currents evoked by hyperpolarizing and depolarizing voltage steps from −100 mV to +100 mV (at V_h_ of −40 mV) were diminished **(** **Fig. 5A** **)**. To elicit TRPV4-mediated currents, astrocytes *in situ* were recorded in Ext2 solution and 10 µM 4αPDD was applied for 6–8 minutes. The cells were clamped at V_h_ of 0 mV and stimulated with a voltage ramp from −100 mV to +100 mV (500 ms) after a 500 ms potential step to −100 mV. Only 25% of astrocytes from controls exhibited 4αPDD-evoked currents, whereas the incidence of responding astrocytes increased 1H (52%) and 7D (59%) after H/I **(** **Fig. 5B** **)**. The threshold for a 4αPDD current response was 120% of the control ramp current (prior to agonist application), at a voltage of +/−100 mV. In response to 4αPDD application, astrocytes exhibited an increase in outward and inward currents **(** **Fig. 5C** **)**, delayed 1–3 minutes after the onset of agonist application, with a rather linear current-voltage relationship **(** **Fig. 5D** **).** Nevertheless, the 4αPDD-evoked current amplitudes were not significantly different when compared to those in control astrocytes or those recorded after H/I **(Fig. S2)**. Exposure to 4αPDD shifted the reversal potential from −15.1±2.2 mV to −5.9±1.7 mV (ΔE_rev_  = 9.1±1.7 mV, n = 13), which is consistent with the development of a cationic current. In the absence of extracellular Ca^2+^ (Ext2_ØCa_), the amplitude of the 4αPDD–evoked currents was reduced by 68.7±18.3% (n = 4, **Fig. 6A**). Finally, the extracellular application of the non-specific inhibitor of TRP channels Ruthenium Red (RR, 10 µM) [33] or RN1734 (10 µM) reduced the 4αPDD-evoked currents by 73.3±15.7% (n = 4) and 56.9±15.0% (n  = 4), respectively **(** **Fig. 6B, C** **).** This finding is a further strong indication of the involvement of TRPV4 in the current response to 4αPDD. This pharmacological profile was qualitatively identical in all three experimental groups. These results overlap those described for the functional expression of TRPV4 in cultured astrocytes [21], and they strongly indicate that TRPV4 channels are also functionally expressed *in situ* in hippocampal astrocytes of the CA1 region and moreover, they are up-regulated after H/I.

**Table 2:** Membrane properties of hippocampal astrocytes recorded in slices from sham-operated (CTRL) rats and 1H and 7D after H/I.

	V_rest_ (mV)	IR (MΩ)	C_m_ (pF)	n
**CTRL**	−75.3±1.2	65.1±9.7	25.8±3.7	16
**1H H/I**	−72.3±1.4	66.8±6.7	24.9±3.6	16
**7D H/I**	−69.1±1.5**	89.4±9.6	20.4±2.6	14

The values are presented as mean ± S.E.M. Asterisks (**p<0.01) indicate very significant differences between astrocytes from control and ischemic rats. Abbreviations: resting membrane potential (V_rest_), input resistance (IR), membrane capacitance (C_m_), number of cells (n).

### TRPV4-mediated Activity is Enhanced in Primary Cultured Astrocytes Isolated from the Ischemic Hippocampal CA1 Region

**Figure 7:**
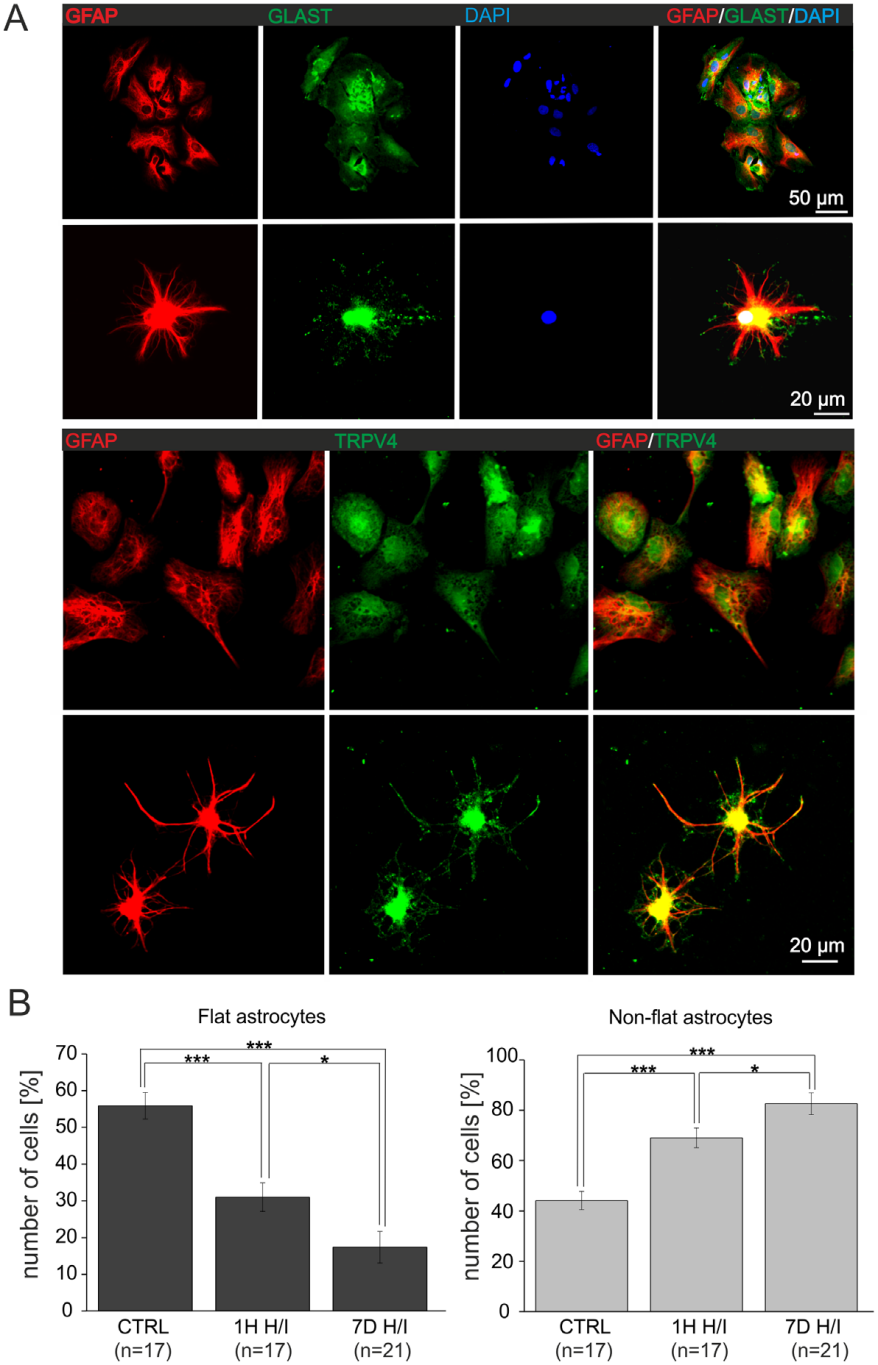
Immunocytochemical identification of cultured astrocytes dissociated from the CA1 region of the hippocampus. (**A**) The distinct morphology of astrocytes isolated from sham-operated rats or from the ischemic hippocampal CA1 region: flat astrocytes (top) and non-flat astrocytes (bottom) cultured for 4–5 days. Both types of astroglial cells expressed GFAP and GLAST and were also positive for TRPV4. (**B**) The incidence of flat and non-flat astrocytes in controls (CTRL) and those isolated from the CA1 hippocampal region 1H and 7D after H/I (n =  number of coverslips). The values are presented as mean ± S.E.M. Statistical significance was calculated using one-way ANOVA. *p<0.05, significant; ***p<0.001, extremely significant.

It is well known that recordings in astrocytes *in situ* are affected by their large passive K^+^ conductance and by their functional coupling to other astrocytes. Moreover, from our *in situ* experiments we could not rule out that the TRPV4-mediated currents were evoked primarily in neurons and, as a result, could then trigger Ca^2+^ entry into astrocytes. To address this issue, we characterized the TRPV4 responses in individual primary astrocytes isolated from the CA1 region of the hippocampus from sham-operated rats and those 1H and 7D after H/I and cultured for 4–5 days. In all experimental groups, immunocytochemical staining of primary cultured astrocytes with antibodies against GFAP and GLAST demonstrated two morphologically distinct types of astrocytes: i.e. astrocytes with a flat polygonal or elongated soma and astrocytes with a non-flat small oval soma and multiple long processes **(** **Fig. 7A** **)**. The numbers of GFAP-positive flat and non-flat astrocytes were determined in controls, 1H and 7D after H/I and expressed as the percentage of the total number of GFAP-positive cells. Interestingly, the proportions of the two morphologically distinct types of astrocytes changed in response to H/I **(** **Fig. 7B** **)**. Astrocyte cultures obtained from control rats comprised 56±3.6% of flat astrocytes (number of coverslips, n = 17), while in cultures obtained from ischemic animals their number significantly declined to 31±3.9% in cultures isolated 1H after H/I (n = 17) and to 17±4.3% in cultures isolated 7D after H/I (n = 21). In contrast, the percentage of non-flat astrocytes increased in response to ischemic injury. Nonetheless, their passive membrane properties were not significantly different from those observed in flat astrocytes.

**Figure 8:**
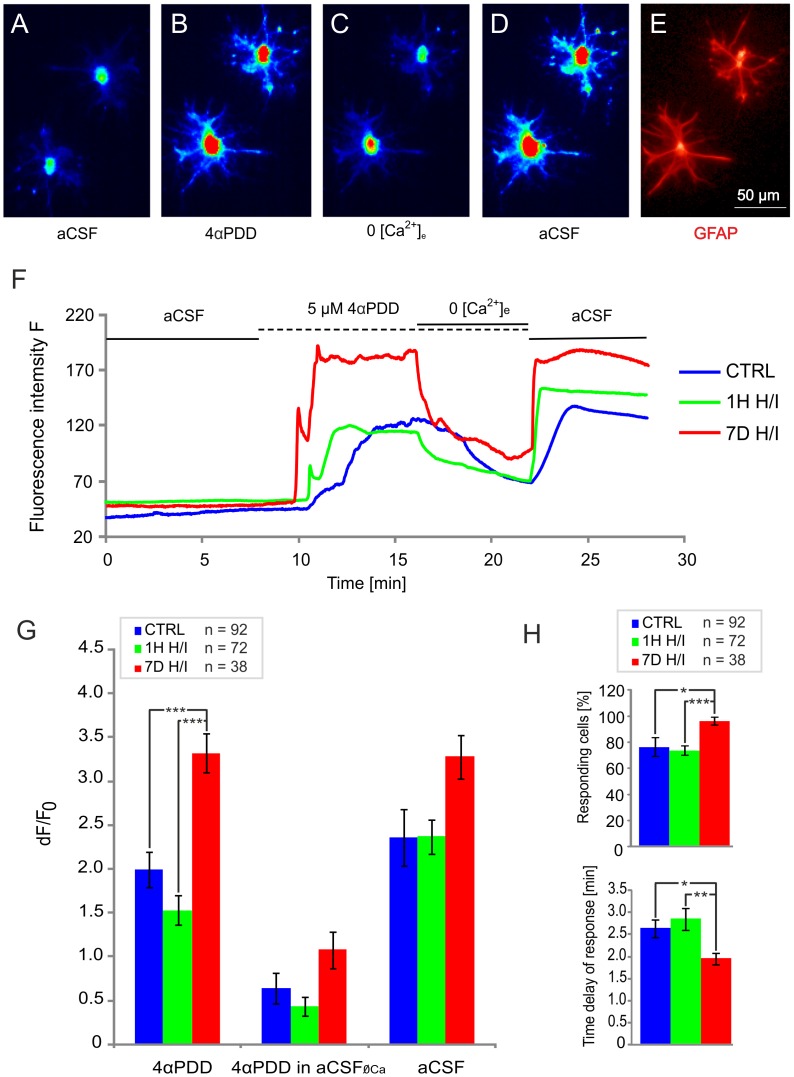
Intracellular Ca^2+^ measurements in cultured astrocytes dissociated from the hippocampal CA1 region. (A–D) Typical fluorescence response elicited by 5 µM 4αPDD in cultured hippocampal astrocytes. (A) Two astrocytes in aCSF before 4αPDD application, (B) during 4αPDD application in aCSF, (C) during 4αPDD application in aCSF_ØCa_ and (D) during washout with aCSF. (E) Immunocytochemical staining for glial fibrillary acidic protein (GFAP) to verify astrocyte identity. (F) Representative fluorescence traces of cultured astrocytes isolated from sham-operated animals (CTRL) and animals 1 hour (1H H/I) and 7 days (7D H/I) after H/I in response to stimulation as in A–D. Note the delay between the 4αPDD challenge and the onset of the fluorescence increase under the 3 conditions. (G) Histogram of the variation in the fluorescence intensities dF/F_0_ depicting the maximum intensity upon 4αPDD application in aCSF (4αPDD), the average intensity during the last minute of 4αPDD application in aCSF_ØCa_ (4αPDD in aCSF_ØCa_) and the maximum intensity during washout (aCSF). (H) Histogram of the number of responding cells (top) and histogram of the 4αPDD response time delay (bottom). Note that in astrocytes 7 days (7D) after H/I, TRPV4-mediated Ca^2+^ entry is enhanced and the number of responding cells is higher when compared to control. The values are presented as mean ± S.E.M. Statistical significance was calculated using one-way ANOVA. *p<0.05, significant; **p<0.01, very significant; ***p<0.001, extremely significant.

The microfluorimetric experiments *in vitro* revealed an increase in [Ca^2+^]_i_ after the application of 5 µM 4αPDD in astrocytes isolated from control animals (n = 92) as well as those 1H (n = 72) and 7D (n = 38) after H/I **(** **Fig. 8A–F** **)**. The [Ca^2+^]_i_ increase was significantly higher in astrocytes isolated from animals 7D after H/I when compared to the other two groups **(** **Fig. 8G** **left columns)**. The Ca^2+^ signal was strongly diminished when 4αPDD was applied in aCSF_ØCa_. The different 4αPDD–mediated Ca^2+^ responses of astrocytes isolated from animals 7D after H/I were also evidenced by the increased number of responding cells **(** **Fig. 8H** **top)** and by the faster onset of response to 4αPDD application **(** **Fig. 8H** **bottom)**. Furthermore, the steepness of the onset of response to 4αPDD was also higher in this group. The differences in these [Ca^2+^]_i_ signal parameters observed in astrocytes were identical in the two morphologically distinct subpopulations.

**Figure 9:**
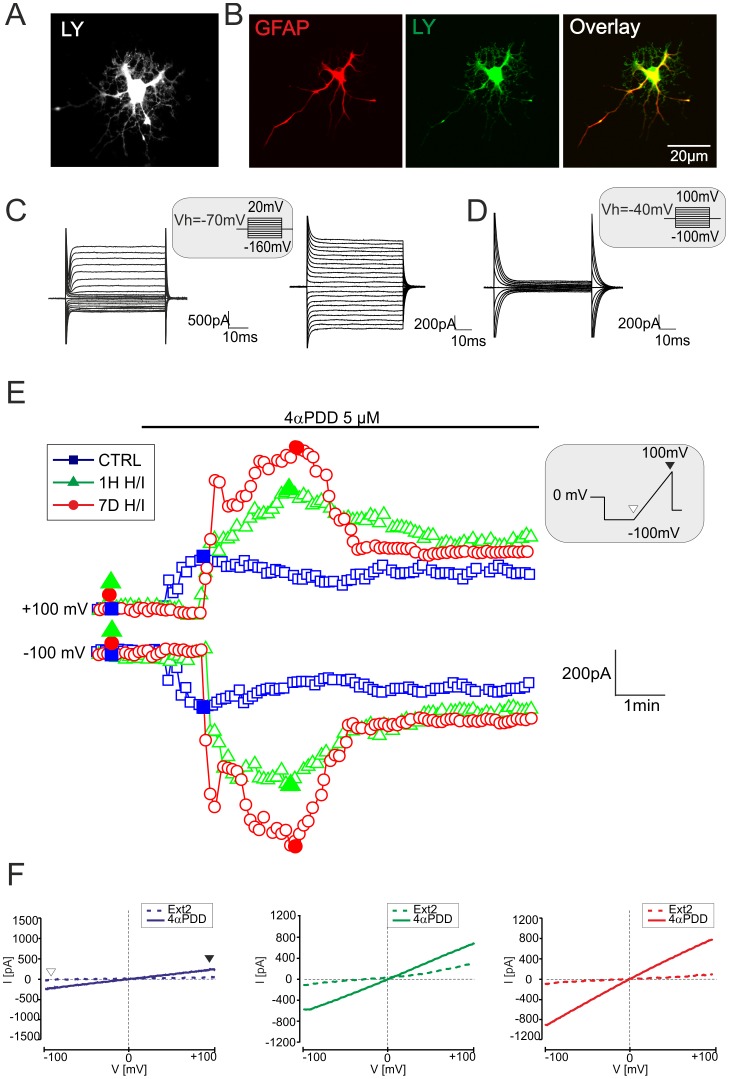
4αPDD-induced currents in cultured astrocytes dissociated from the hippocampal CA1 region. (**A**) An image of a Lucifer Yellow (LY)-loaded astrocyte taken by a digital camera immediately after patch-clamp recording and (**B**) the same astrocyte identified by immunostaining with glial fibrillary acidic protein (GFAP). The overlay image shows the co-localization of GFAP with LY. (**C**) “Complex” and “passive” current patterns in astrocytes *in vitro* evoked by membrane depolarization and hyperpolarization from the holding potential of −70 mV. The currents were recorded using K^+^- and Na^+^-containing intra- and extracellular solutions (Int1 and Ext1). The voltage step protocol is shown in the inset. (**D**) Current pattern evoked with a voltage step protocol (see the inset) in cultured astrocytes recorded in intra- and extracellular solutions in which K^+^ and Na^+^ were replaced by Cs^+^ (Int2 and Ext2). Note the marked reduction in membrane conductance. (**E**) Time course of 4αPDD-evoked currents measured from the ramp protocol in astrocytes of controls (blue squares) and astrocytes 1H (green triangles) and 7D after H/I (red circles). Currents were measured at −100 mV (white arrowhead) and +100 mV (black arrowhead) in response to a voltage ramp stimulation protocol (see the inset). (**F**) Representative traces of steady state currents (same cells as in E) recorded prior to (in Ext2 solution, dashed line) and during 4αPDD application (full line) in cultured astrocytes of controls (left) and those 1H (middle) and 7D after H/I (right). Representative traces of steady state currents were obtained at the times indicated by the filled blue squares, green triangles and red circles in E. White and black arrowheads indicate the applied voltage ramp and the corresponding current traces (see the inset in E).

In order to identify the current pattern of the two types of astrocytes *in vitro*, physiological Na^+^- and K^+^-containing solutions were used (**Table 1**). The cells labeled with LY or Alexa-Fluor hydrazide during patch clamp recording were identified as astrocytes using an antibody against GFAP **(** **Fig. 9A, B** **)**. The membrane currents of astrocytes were recorded in response to hyperpolarizing and depolarizing voltage steps, ranging from −160 mV to +20 mV at V_h_ of −70 mV. Isolated astrocytes cultured for 4–5 days showed two distinct current profiles as described on freshly isolated astrocytes by Zhou and Kimelberg [34]. The flat astrocytes were mainly characterized by passive K^+^ currents (**Fig. 9C** **right)**, whereas non-flat astrocytes showed a “complex” current profile **(** **Fig. 9C** **left)**. Since the passive membrane properties of flat and non-flat astrocytes in each experimental group were not significantly different, they were pooled together (**Table 3** **).** Also microfluorimetric measurements did not reveal any differences in 4αPDD-evoked [Ca^2+^]_i_ signals between flat and non-flat astrocytes in each experimental group, therefore both types of astrocytes were used for the electrophysiological analyses.

**Table 3:** Membrane properties of isolated astrocytes from the CA1 region of the hippocampus from sham-operated (CTRL) rats and 1H and 7D after H/I.

	V_rest_ (mV)	IR (MΩ)	C_m_ (pF)	n
**CTRL**	−71.3±1.9	296.3±78.2	18.5±2.4	9
**1H H/I**	−77.2±1.4	211.8±58.8	26.6±1.2**	25
**7D H/I**	−71.3±2.7	265.3±55.0	19.7±2.7	8

The values are presented as mean ± S.E.M. Asterisks (**p<0.01) indicate very significant differences between astrocytes from control and ischemic rats. Abbreviations: resting membrane potential (V_rest_), input resistance (IR), membrane capacitance (C_m_), number of cells (n).

**Figure 10:**
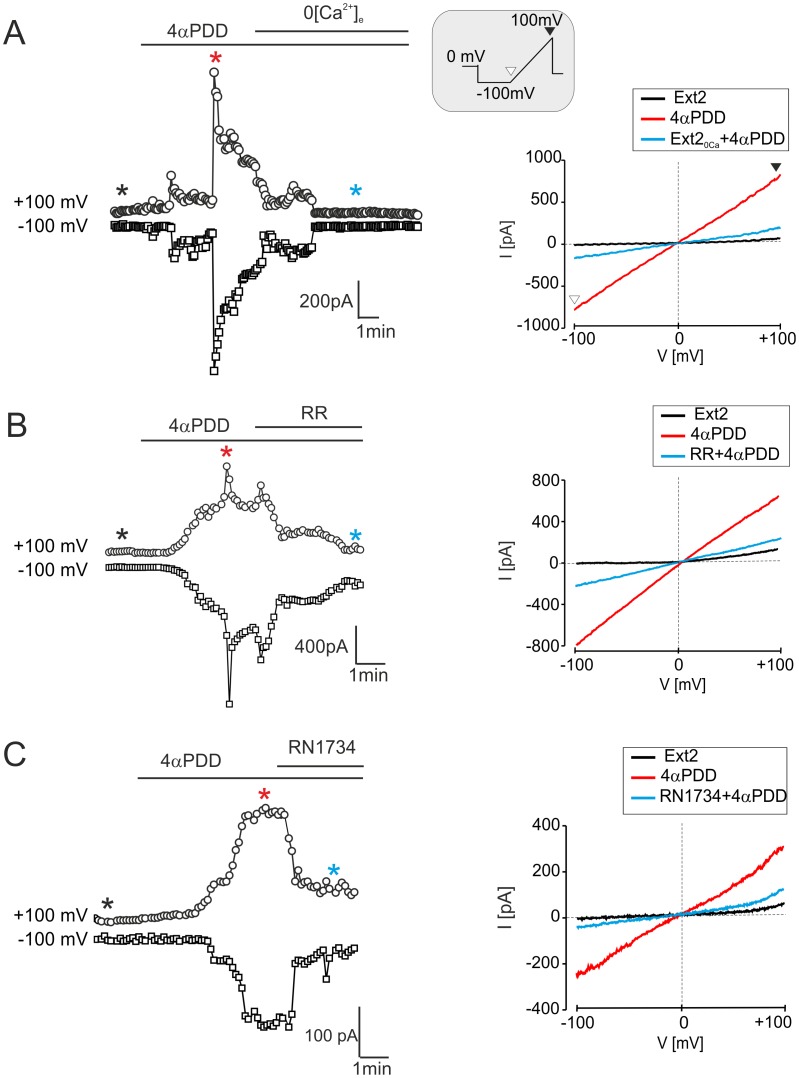
Currents evoked by 4αPDD in astrocytes *in vitro* are reduced by calcium-free extracellular solution, Ruthenium Red or RN1734. (**A–C, left**) Time course of 4αPDD-evoked currents measured from the ramp protocol in astrocytes isolated from the hippocampus 7D after H/I (for the voltage protocol see the inset) prior to and during 4αPDD (5 µM) application and after removing extracellular Ca^2+^ (Ext2_ØCa_, **A**) or after the application of TRPV4 inhibitors, such as Ruthenium Red (RR, 10 µM, **B**) or RN1734 (10 µM, C). (A–C, right) The traces of steady state currents (same cells as in left) obtained in Ext2 solution (black lines), during 4αPDD application (red lines) and after removing extracellular Ca^2+^ (Ext2_ØCa_, **A**) or after the application of TRPV4 inhibitors, such as Ruthenium Red (RR, 10 µM, **B**) or RN1734 (10 µM, **C**), are indicated by blue lines. Representative traces of steady state currents were obtained at the times indicated by asterisks of the corresponding colors.

**Figure 11:**
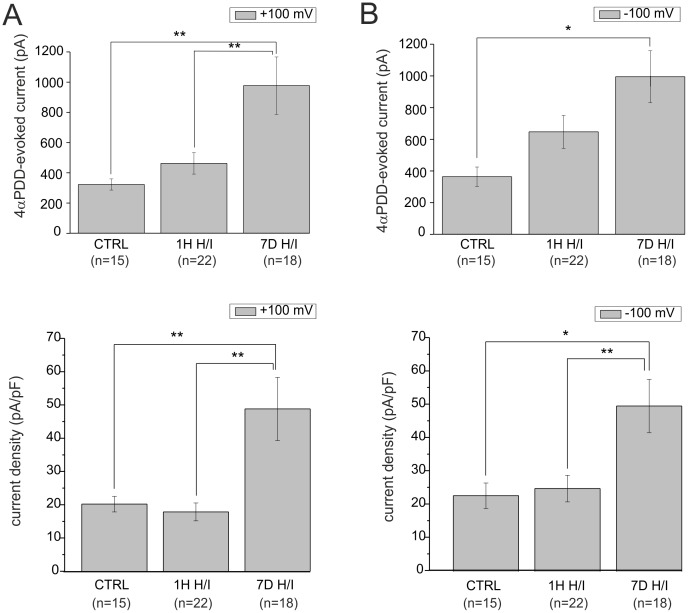
Changes in TRPV4-mediated currents in response to hypoxia/ischemia. Histograms of 4αPDD-evoked changes in current amplitudes **(A top)** and current densities **(A bottom)** at +100 mV and current amplitudes **(B top)** and current densities **(B bottom)** at −100 mV in control astrocytes (CTRL), and those isolated from rats 1H and 7D after H/I. The values are presented as mean ± S.E.M. Statistical significance was calculated using one-way ANOVA. *p<0.05, significant; **p<0.01, very significant.

Similarly to the *in situ* experiments, TRPV4-specific cationic current recordings were performed using intra- and extracellular solutions in which K^+^ and Na^+^ were replaced with cesium. Under these experimental conditions, the membrane currents evoked by hyperpolarizing and depolarizing voltage steps from −100 mV to 100 mV were strongly diminished **(** **Fig. 9D** **).** The application of 4αPDD (5 µM) produced an increase in membrane conductance in the astrocytes of controls as well as in those 1H and 7D after H/I when stimulated with a voltage ramp from −100 mV to +100 mV from V_h_ of 0 mV **(** **Fig. 9E** **)**. Exposure to 4αPDD caused a positive shift of E_rev_ from −5.8±1.1 mV to −1.1±0.9 mV (ΔE_rev_  = 4.7±0.9 mV, n = 19). The increase in ramp current upon 4αPDD application was diminished by 74.0±11.9% (n = 6) by omitting [Ca^2+^]_e_ and was depressed by RR (10 µM) or RN1734 (10 µM) by 79.7±10.5% (n = 8) and by 69.8±18.7% (n = 8), respectively **(** **Fig. 10 A–C** **).** Of note, the 4αPDD-evoked outward and inward current amplitudes and densities were significantly augmented in astrocytes isolated from the rat hippocampus 7D after H/I **(** **Fig. 11** **)**.

Collectively, these results support the tenet that TRPV4 channels are expressed by CA1 primary astrocytes in culture and that their activity is significantly up-regulated in astrocytes isolated from the ischemic CA1 region seven days after H/I.

## Discussion

In the present study, we provide the first evidence of the functional expression of TRPV4 channels in CA1 hippocampal astrocytes *in situ*. Moreover, this is the first study describing the impact of global cerebral ischemia *in vivo* on TRPV4-mediated currents and intracellular Ca^2+^ signaling in hippocampal astrocytes. We found that the activity of TRPV4 channels markedly increases in astrocytes in the CA1 region of the adult rat hippocampus during the development of astrogliosis induced by hypoxic/ischemic injury.

It has been shown previously that TRPV4 is widely expressed in the brain, particularly in the neurons of the pyramidal hippocampal layer as well as in cortical and hippocampal astrocytes [9], [16], [21]. However, there are no data demonstrating the functional expression of TRPV4 channels in astrocytes *in situ* under either physiological or pathophysiological conditions, such as ischemia. In agreement with previous studies [8], [16], we showed the expression of TRPV4 protein in hippocampal pyramidal neurons as well as in astrocytes of the CA1 region of the hippocampus in control rats. In addition, we found that TRPV4 immunoreactivity increases in hippocampal astrocytes starting 1 hour after ischemia and reaches maximal levels after 7 days of reperfusion. Our immunohistochemical analyses revealed that CA1 pyramidal neurons express TRPV4 in both sham-operated rats and rats 1 hour after H/I, but not 7 days after H/I. This decrease in neuronal TRPV4 expression over time is clearly due to extensive apoptosis in the hippocampal CA1 region wiping out the majority of CA1 pyramidal neurons [22]. Hippocampal astrocytes in sham-operated animals showed very low TRPV4 expression, primarily detected in astrocytic processes enwrapping blood vessels. It is noteworthy that TRPV4 immunoreactivity markedly increased 1 hour and 7 days after H/I. Our Western blot analyses revealed an increase in total TRPV4 protein content 1 hour after ischemia and, moreover, quantitative RT-PCR analyses detected a ∼ 2.4-fold increase in TRPV4 mRNA. A similar finding of a rapid increase in astroglial acetylcholinesterase (AChE) gene expression (a two- to three-fold increase in readthrough-AChE within 1 h post-ischemia) has been described previously [35]. These authors suggested that the stress-induced increase in AChE release is due to the up-regulation of functional readthrough-AChE isoform expression, although it is possible that this increased release of AChE 1 hour post-ischemia may be attributed to increased readthrough-AChE mRNA transcript stability as well as to an increase in the rate of AChE transcription. Additionally, the increase in TRPV4 protein levels observed 1H after H/I might be, at least partially, due to the enhancement of TRPV4 post-transcriptional modifications occurring within hours after hypoxic/ischemic stress. This could lead to the appearance of additional bands after 1H and 6H corresponding to different isoforms of TRPV4, previously detected in astrocytes and shown by Benfenati and co-authors [21]. Nevertheless, we hypothesize that slower TRPV4 protein degradation or protein stabilization might also contribute to the increased amount of TRPV4 protein 1H after H/I as well.

Curiously, a decline in the total content of TRPV4 protein was observed starting 1 day after H/I. Since at this time point neurons are no longer present in the CA1 region of the hippocampus due to massive neuronal cell death, a decline in the levels of TRPV4 protein in the hippocampal CA1 region is likely to occur, hence the remaining expression of TRPV4 protein is almost exclusively in reactive astrocytes. Of note, the enhanced TRPV4 expression coincided with maximal GFAP expression, astrocyte hypertrophy and increased astrocytic proliferation [22], which might suggest that TRPV4-mediated Ca^2+^ signaling plays an important role in the formation of the adaptive response of the gliotic scar. However, we cannot rule out that increased TRPV4 activity might be also linked to the initiation of delayed astrocytic cell death as described by Cao and co-authors [36].

Considering that the physiological role of TRPV4 is mainly related to its ability to permeate Ca^2+^ [37], any investigation of the functional expression of TRPV4 in astrocytes *in situ* must include an analysis of the role of TRPV4 in astroglial [Ca^2+^]_i_ signaling. In control astrocytes, we have recorded low frequency of spontaneous transient [Ca^2+^]_i_ oscillations, while in astrocytes 1 hour and 7 days after H/I, these oscillations increased 7- and 20-fold, respectively. Under physiological conditions, similar low frequency of spontaneous transient [Ca^2+^]_i_ oscillations mediated mainly by the release of Ca^2+^ from internal stores were demonstrated in astrocytes *in situ* [38]. During cerebral hypoxia/ischemia, it is known that astrocyte damage can be evoked by a rapid increase in [Ca^2+^]_i_ [2], a result in agreement with our data demonstrating augmented spontaneous [Ca^2+^]_i_ transients in ischemic astrocytes *in situ*. Our results also accord well with previous data showing enhanced spontaneous [Ca^2+^]_i_ oscillations in other models of ischemic injury in rodents (for a review see [39]), in human astrocytes of epileptic patients [40] and under pathophysiological conditions, such as Rasmussen’s encephalitis [41]. Since the expression of a functional NMDA receptor subtype 2B was described in astrocytes following ischemia *in vivo* and anoxia *in vitro* [42], we cannot rule out the possibility that the increased spontaneous Ca^2+^ oscillations in post-ischemic astrocytes in slices might be partially due to arachidonic acid-mediated potentiation of the current through the NMDA receptor channels, as previously demonstrated in cerebellar granule cells [43]. Nevertheless, blocking spontaneous Ca^2+^ oscillations with RN1734 confirmed that these oscillations are also partially mediated by TRPV4 channels. The duration of reperfusion following H/I also altered the [Ca^2+^]_i_ dynamics. In control astrocytes and those 1H after H/I, mainly an increased frequency of [Ca^2+^]_i_ transients was observed, while 7 days following ischemia we also detected sustained Ca^2+^ entry after 4αPDD application in 57% of astrocytes, which was completely blocked by Ca^2+^-free extracellular solution **(** **Fig. 3** **)**; however, it was insensitive to the specific TRPV4 inhibitor RN1734 **(** **Fig. 4** **)**. In controls and 1H after H/I, only 3 and 15% of astrocytes showed sustained Ca^2+^ entry, respectively. We hypothesize that TRPV4-mediated Ca^2+^ entry could initiate an additional Ca^2+^ influx via the activation of other membrane proteins, such as sodium-calcium exchangers (NCX) [44]. These authors showed that hypoxia also induces Ca^2+^/Na^+^ influx via TRPC1, 3, 6 channels, resulting in the reversal of NCX and thus to an elevation of intracellular Ca^2+^ in PC12 cells. TRPV1-TRPV4 are non-selective cationic channels, moderately permeable to Ca^2+^, with a permeability ratio (*P*_Ca_ */P*_Na_) between ∼6–10 [45]. Such a hypothesis is supported by data demonstrating that the reverse mode of NCX contributes to astrocyte and neuronal pathology following experimental traumatic brain injury [46]. NCX-mediated fluxes of Ca^2+^ in both forward and reverse modes were found in astrocytes *in vitro* as well as those *in situ* [47], [48] and moreover, the activation of astroglial ionotropic receptors or glutamate transporters induced a marked elevation of intracellular Na^+^, which also switched the exchanger to the reverse mode [47], [49], [50]. The reversal of NCX could be also triggered by mild depolarization [51], which occurs in reactive astrocytes due to Kir4.1 down-regulation [23].

The question arises: what is the role of TRPV4 in the augmented [Ca^2+^]_i_ signaling found in astrocytes after ischemia. The possible implication of TRPV4 in enhancing the frequency of Ca^2+^ oscillations in astrocytes after H/I is plausible. In previous studies, a potential role for TRP channels in a pathophysiological [Ca^2+^]_i_ rise in neurons has been suggested [52]–[55]. In particular, TRPM7 and thermal-sensitive TRPV3 have been demonstrated to mediate a post-ischemic [Ca^2+^]_i_ elevation that ultimately leads to neuronal death [9]. Our data demonstrate that increased spontaneous [Ca^2+^]_i_ oscillations in astrocytes 1 hour and 7 days after hypoxia/ischemia are paralleled by a 2- to 3-fold increase in 4αPDD-induced Ca^2+^ influx. Our finding that spontaneous Ca^2+^ oscillations are partially carried by TRPV4 channels in astrocytes 7D after H/I contrasts with a large body of evidence documenting the involvement of Ca^2+^ release from intracellular stores in astrocytic Ca^2+^ oscillations rather than Ca^2+^ influx through the membrane [39]. TRPV4 is a polymodal channel that is activated/modulated by different intracellular messengers comprising IP_3_ [56], phosphorylation [57], changes in external and internal Ca^2+^ concentrations, PLC activity, and AA metabolites [31], which are generated via a PLA2 pathway. Of note, in native ciliated epithelial cells, the IP_3_-mediated sensitization of TRPV4 positively modulates its ability to act in response to osmotic stress via epoxyeicosatrienoic acid, a known PLA2 secondary messenger [56]. The latter signaling pathway is known to be up-regulated after ischemia and involved in augmented [Ca^2+^]_i_ oscillations. Intriguingly, in the same study functional coupling to TRPV4 and intracellular Ca^2+^ stores via IP_3_ signaling was shown to trigger oscillatory [Ca^2+^]_i_ signals in native ciliated epithelial cells [56]. The involvement of TRPV4 in increasing spontaneous [Ca^2+^]_i_ rises in astroglia after an ischemic insult is further corroborated by the observation that the specific TRPV4 activator 4αPDD increased the frequency of [Ca^2+^]_i_ transients and promoted sustained Ca^2+^ entry 7 days after H/I.

Another explanation for TRPV4 up-regulation could originate from the proposed role of TRPV4 as mechanosensor or osmosensor in different cell types, including astrocytes [18], [58]. In the present study we showed that not all hippocampal astrocytes responded to TRPV4-agonist in control rats, but the number of responding cells significantly increased after ischemia (**Fig. 3** **,** **5**). Since our previous studies demonstrated that astrocytes *in situ* respond differently to hypo-osmotic stress, a condition simulating cell swelling frequently observed after ischemia [59], or to oxygen-glucose deprivation [60], it could be envisaged that under physiological conditions only the subpopulation of astrocytes that is more capable of cell volume regulation expresses TRPV4. Therefore, the up-regulation of TRPV4 after ischemia could be linked to a required increase in the control of astrocytic volume under hypoxia/ischemia, a pathological state characterized by impaired glial volume homeostasis [61]. In addition, astrocytes release various bioactive substances via Ca^2+^- and soluble N-ethylmaleimide-sensitive factor attachment protein receptor (SNARE)-dependent exocytosis, which functions as the main astroglial pathway in glia-neuron signaling [62]–[64]. Thus, we suggest that the increased expression/activation of TRPV4 channels in reactive astrocytes might also result in a Ca^2+^-dependent release of cytokines or growth factors. Nevertheless, the mechanisms of exocytosis in astrocytes after ischemia are still poorly understood, and further studies using transgenic TRPV4-deficient mice could potentially clarify the role of this polymodal sensor in ischemic astrocytes.

Our study clearly shows that the increase in TRPV4-mediated [Ca^2+^]_i_ signaling was positively correlated with the time of reperfusion following H/I. An alteration in the activation of TRPV4 channels, possibly via a calmodulin (CaM) binding site or PLA2 activation pathways as described previously [31], [65], could be involved. Indeed, the activity of these enzymes is known to be increased during hypoxia [66]. However, the increased TRPV4 current amplitudes and [Ca^2+^]_i_ signals were paralleled by increased immunostaining for TRPV4 in astrocytes in the CA1 region of the hippocampus, thereby suggesting that the elevation in protein expression could account, at least partially, for the up-regulation of the TRPV4-mediated [Ca^2+^]_i_ signals observed after ischemia.

Despite the fact that our *in situ* experiments showed TRPV4-specific Ca^2+^ entry in astrocytes, one can argue that these Ca^2+^ transients might be evoked indirectly by the activation of TRPV4 in hippocampal neurons. It is in fact well established that astrocytes respond to enhanced neuronal activity by a rise in intracellular Ca^2+^ [67]. However, TRPV4-mediated currents/Ca^2+^ entry markedly increased when the neuronal loss was maximal, e.g. 7 days after ischemia, while 1 hour after ischemia the changes were less pronounced. However, the selective *in vitro* characterization of astroglial TRPV4-responses in adult primary astrocytes isolated from sham-operated as well as ischemic rats confirmed that both TRPV4-mediated currents and Ca^2+^ entry were astrocyte-specific and displayed a similar behavior as those *in situ*. Notably, the analyses we performed in astrocytes *in vitro* confirmed that 4αPDD-evoked Ca^2+^ entry was due to the direct activation of TRPV4 on the astrocytic membrane and showed that 4αPDD-evoked Ca^2+^ entry is also markedly increased 7 days after H/I. Finally, we observed an increase in 4αPDD current *in situ*, in cells that had been counterstained either with Alexa Fluor hydrazide or LY and that we had clearly identified as astrocytes by immunohistochemistry.

Our electrophysiological data are in agreement with the previous pharmacological characterization of TRPV4-mediated currents in astrocytes *in vitro* as well as that carried out in heterologous expression systems with respect to TRPV4 channel sensitivity to extracellular Ca^2+^ decrease and Ruthenium Red inhibition [21]. Our data are also comparable with the pharmacological properties of TRPV4-specific currents described in hippocampal neurons [16]. Here, we also used a new TRPV4-specific antagonist, RN1734, which blocks 4αPDD-evoked currents [32]. Interestingly, the 4αPDD-evoked currents of adult astrocytes *in situ* **(** **Fig. 5D** **)** as well as those *in vitro* **(** **Fig. 9F** **)** displayed a rather linear current-voltage relationship, similar to that observed in striatal neurons and HEK293 cells transiently expressing rat TRPM2 [68]; however, these channels are not expressed in astrocytes [8]. Even performing point-to-point digital subtraction of the current traces revealed a rather linear or weakly rectifying current-voltage relationship of 4αPDD-currents in astrocytes *in situ* and *in vitro* (**Fig. S3).** A similar current-voltage relationship was demonstrated in mutated TRPV4 channels [69], in which both aspartates in the TRPV4 pore region were neutralized, causing a marked reduction in outward rectification. Moreover, a modest outward/inward rectification was also shown in HEK293 cells expressing human TRPV4 [70] and in human airway epithelial CFT1-LCFSN cells [71]. Since it is not obvious what underlies the linear TRPV4 current-voltage relationship in adult astrocytes, we hypothesize that post-translational modifications, conformational changes of the TRPV4 protein or protein-protein interactions might contribute to such current behavior.

Generally, we observed marked heterogeneity in TRPV4 channel activity during 4αPDD application even within astrocytes from sham-operated animals or those after H/I. Heterogeneous responses of astrocytes to 4αPDD application were described previously [21]and also recently, by Lanciotti and co-authors [72]. Both groups demonstrated marked differences in the activation/inactivation of TRPV4–mediated Ca^2+^ entry and the response onset within the recorded population of astrocytes. Such diversity in TRPV4 channel activity could possibly originate from alterations in extracellular/intracellular Ca^2+^ in the vicinity of the astrocytic membrane, as these have been shown to strongly regulate TRPV4 channels [31], [45]. In some astrocytes, 4αPDD-evoked currents displayed either fast activation and fast inactivation, which have been reported to occur in high extracellular Ca^2+^ concentrations [31], or a slow rate of current decay, which might be attributed to [Ca^2+^]_i_ levels. Furthermore, an interaction between the C- and N-termini of the TRPV4 channel was found to control the Ca^2+^-dependent potentiation of the channel [73], and the presence of different splice variants, their homomerization/heteromerization or post-translational modifications of the TRPV4 protein might also contribute to the diversity in TRPV4 channel activation/desensitization following H/I. Until now, there are no published data describing TRPV4 splice variants in rats; however, the existence of 5 different splice variants was described for the human TRPV4 channel [74]. Moreover, 4αPDD might act on the lipid environment more efficiently *in vitro* than *in situ* and thus differentially modulate TRPV4 channels. The physiological relevance of such a lipid-modulated mechanism of activation has been suggested for other mechano-gated channels [75]. Finally, astrocyte heterogeneity within the hippocampal CA1 region might be the reason for the observed variations in TRPV4 responses. A number of publications have appeared recently showing marked astrocyte heterogeneity even within an individual CNS region, with different astrocyte coupling or the expression of ion channels, glutamate transporters or receptors [29], [60], [76]. Moreover, the reactive astrocytes in post-ischemic tissue might originate not only from proliferating astrocytes [22], but also from polydendrocytes [77], which might further contribute to the heterogeneity within the reactive astrocyte population.

In agreement with our Ca^2+^ imaging data, electrophysiological analysis also confirmed that after H/I astrocytes display increased TRPV4 currents**.** These changes in TRPV4 current amplitude were not significant *in situ* **(Fig. S2).** However, this result is not surprising because the expression of passive conductance in astrocytes *in situ* makes the isolation of different currents in these cells quite difficult [23]. Importantly, we found that TRPV4-mediated currents in astrocytes *in vitro* are significantly increased 7 days after H/I; however, 4αPDD-evoked Ca^2+^entry/currents were not enhanced in astrocytes isolated from the hippocampal CA1 region 1H after H/I. We hypothesize that astrocytes isolated during the acute stages of reperfusion could recover after their transfer into culture medium and therefore their responses to 4αPDD were similar to those recorded in controls. In contrast, 7D after H/I the changes in reactive astrocytes are rather permanent and therefore not reversible by their culturing.

In addition, we found that the incidence of astrocytes responding to 4αPDD increases with the time of reperfusion and that the TRPV4-immunoreactivity of an individual reactive astrocyte in the CA1 region significantly increases, correlating well with the increasing number of TRPV4-positive astrocytes in the hippocampal CA1 region during reperfusion (**Fig. 1** **,** **3H** **,** **5B** **,** **8H**). Although we were not able to detect a significant increase in TRPV4-current amplitude *in situ* following ischemia, intracellular Ca^2+^ imaging revealed that Ca^2+^ entry mediated by TRPV4 channels is significantly augmented in astrocytes 1 H and 7D after H/I. A significantly larger number of cells was analyzed by intracellular calcium imaging compared to patch-clamp recording *in situ*, which might result in the discrepancies between TRPV4-mediated Ca^2+^ entry and current amplitude. In contrast to intracellular Ca^2+^ measurements, where relatively intact cells are recorded, the patch-clamp technique in the whole-cell configuration significantly affects the astrocytic intracellular environment, possibly diluting the regulatory molecules required for enhanced TRPV4 activity.

Collectively, the data obtained *in vitro* validate the *in situ* analyses, thus identifying TRPV4 as one of the channels involved in the pathophysiological [Ca^2+^]_i_ signals in ischemic astroglia. Nevertheless, it still remains uncertain how astrocytic TRPV4 channels are activated under physiological or pathophysiological conditions. Generally, astrocytes play a significant role in ionic/neurotransmitter and water homeostasis and therefore, neuronal activity leading to enhanced K^+^/glutamate uptake in astrocytes and resulting in the swelling of astrocytic processes enwrapping the synapses might be an initial trigger for TRPV4 activation, as these channels act as mechanosensors. Also, subtle changes in extracellular ionic concentrations in the vicinity of the astrocytic membrane might result in TRPV4 activation under physiological conditions, as these channels act as osmosensors. Recent data indicate that astrocytes contain a TRPV4/AQP4 complex [18] that constitutes a key element in CNS volume homeostasis by acting as an osmosensor that couples osmotic stress to downstream signaling cascades. Additionally, the fact that several ion channels, such as Kir channels (specifically Kir4.1), AQP4 and TRPV4, are co-expressed in the astrocytic endfeet [78]–[80] points to the importance of membrane protein interactions of membrane microdomains that are devoted to extracellular K^+^ buffering/glutamate uptake and water homeostasis. Thus, astrocytic swelling via PLA_2_ activation, the release of AA from membrane phospholipids followed by cytochrome P450 epoxygenase-dependent AA metabolism producing 5′,6′ epoxyeicosatrienoic acid might act as a TRPV4 channel activator. Under pathophysiological conditions, tissue damage and inflammation result in the release of pro-inflammatory mediators, which may in turn activate intracellular signaling pathways and downstream kinases, such as PKC and PKA, and consequently enhance the activation the TRPV4 ion channel by phosphorylation [57].

Taken together, we show that TRPV4 channels are involved in the increase of [Ca^2+^]_i_ signaling occurring after cerebral hypoxia/ischemia in the hippocampal astrocytes of adult rats. The TRPV4-mediated [Ca^2+^]_i_ elevation is at least partially due to the increased expression of TRPV4 protein and is further increased with time following the ischemic insult. Because alterations in [Ca^2+^]_i_ dynamics in astroglia are associated with the modulation of physiological and pathophysiological processes (for a review see [81], [82]), our study identifies a novel molecular target to be explored in order to unravel astrocyte signaling after ischemia reperfusion and to define more clearly the astrocyte-mediated pathogenetic processes in acute brain disorders.

## Supporting Information

Figure S1 **Quantification of TRPV4 protein levels in the hippocampal CA1 region following hypoxia/ischemia. (A)** Western blots demonstrating the changes in TRPV4 and GFAP protein levels in response to hypoxic/ischemic (H/I) injury. β-actin was used as a control for sample loading. Squares A1-6 (red) and A1’-6′ (blue) indicate the zones where TRPV4 quantification was carried out, containing 1 or 2 TRPV4 bands, respectively. Squares B1-6 (green) and C1-6 (yellow) indicate the zones where GFAP and β-actin quantification was carried out, respectively. The effect of H/I on TRPV4 levels during the time of reperfusion was evaluated in each individual Western blot and expressed as the percent increase/decrease related to the TRPV4 content in control samples (CTRL), which was set as 100%. To obtain the area corresponding to the TRPV4 protein level at each time-point, the area A1/A1’ (TRPV4) was divided by the area C1 (β-actin) and this value was set as 100%. **(B)** Single Western blot quantification that demonstrates an increase in TRPV4 protein levels 1H after H/I regardless of the selected zones (A1-6 or A1’-6′) and an increase in GFAP content 3 and 7 days (3D, 7D) after H/I.
(TIF)

Figure S2 **Changes in TRPV4-mediated currents in astrocytes** ***in situ*** **following hypoxia/ischemia.** Histograms of 4αPDD-evoked changes in current amplitudes at +100 mV **(left)** and -100 mV **(right)** in control astrocytes (CTRL) and astrocytes in slices prepared from rats 1H and 7D after H/I.
(TIF)

Figure S3 **Isolation of 4αPDD currents –point-to-point current subtraction.** The 4αPDD- current traces obtained after point-to-point subtraction of ramp current traces recorded prior to 4αPDD application *(a)* and those recorded during 4αPDD application *(b)* in astrocytes *in situ* **(A)** and astrocytes isolated from the adult rat hippocampal CA1 region **(B)** of sham-operated rats (CRTL) and rats 1 hour and 7 days after hypoxia/ischemia (1H, 7D H/I; see the voltage ramp protocol in the inset). Note the linear I-V relationship of the 4αPDD-sensitive current *(b-a)* detected in astrocytes *in situ* as well as *in vitro* in sham-operated rats (blue trace), while a modest outward rectification of the 4αPDD current was observed in post-ischemic astrocytes (red and green traces). Typical 4αPDD-current traces obtained after point-to-point subtraction of ramp current traces recorded in astrocytes *in situ* **(C)** and astrocytes *in vitro* **(D)** prior to *(a)* and during 4αPDD application *(b)* and in response to the removal of extracellular Ca^2+^ (Ext2_ØCa_, top) or after the application of TRPV4 inhibitors, such as 10 µM RN1734 (middle) or 10 µM,ruthenium red (RR, bottom, *c*). Red traces represent the 4αPDD-sensitive current *(b-a)* and blue traces represent the remaining current after applying the inhibitors *(c-a)*. Black and white arrowheads indicate the applied voltage protocol (see the insets) and the corresponding current traces.
(TIF)

## References

[1] Chvatal A, Anderova M, Neprasova H, Prajerova I, Benesova J (2008). Pathological potential of astroglia.. Physiol Res.

[2] Verkhratsky A, Anderova M, Chvatal A (2009). Differential calcium signalling in neuronal-glial networks.. Front Biosci.

[3] Nedergaard M, Rodriguez JJ, Verkhratsky A (2010). Glial calcium and diseases of the nervous system.. Cell Calcium.

[4] Latour I, Hamid J, Beedle AM, Zamponi GW, Macvicar BA (2003). Expression of voltage-gated Ca2+ channel subtypes in cultured astrocytes.. Glia.

[5] Lalo U, Pankratov Y, Kirchhoff F, North RA, Verkhratsky A (2006). NMDA receptors mediate neuron-to-glia signaling in mouse cortical astrocytes.. J Neurosci.

[6] Cotrina ML, Nedergaard M (2009). Physiological and pathological functions of P2X7 receptor in the spinal cord.. Purinergic Signal.

[7] Matsuda T, Arakawa N, Takuma K, Kishida Y, Kawasaki Y (2001). SEA0400, a novel and selective inhibitor of the Na+-Ca2+ exchanger, attenuates reperfusion injury in the in vitro and in vivo cerebral ischemic models.. J Pharmacol Exp Ther.

[8] Bai JZ, Lipski J (2010). Differential expression of TRPM2 and TRPV4 channels and their potential role in oxidative stress-induced cell death in organotypic hippocampal culture.. Neurotoxicology.

[9] Lipski J, Park TI, Li D, Lee SC, Trevarton AJ (2006). Involvement of TRP-like channels in the acute ischemic response of hippocampal CA1 neurons in brain slices.. Brain Res.

[10] Cao DS, Yu SQ, Premkumar LS (2009). Modulation of transient receptor potential Vanilloid 4-mediated membrane currents and synaptic transmission by protein kinase C. Mol Pain.

[11] Golovina VA (2005). Visualization of localized store-operated calcium entry in mouse astrocytes. Close proximity to the endoplasmic reticulum.. J Physiol.

[12] Malarkey EB, Ni Y, Parpura V (2008). Ca2+ entry through TRPC1 channels contributes to intracellular Ca2+ dynamics and consequent glutamate release from rat astrocytes.. Glia.

[13] Shirakawa H, Sakimoto S, Nakao K, Sugishita A, Konno M (2010). Transient receptor potential canonical 3 (TRPC3) mediates thrombin-induced astrocyte activation and upregulates its own expression in cortical astrocytes.. J Neurosci.

[14] Kauer JA, Gibson HE (2009). Hot flash: TRPV channels in the brain.. Trends Neurosci.

[15] Guler AD, Lee H, Iida T, Shimizu I, Tominaga M (2002). Heat-evoked activation of the ion channel, TRPV4.. J Neurosci.

[16] Shibasaki K, Suzuki M, Mizuno A, Tominaga M (2007). Effects of body temperature on neural activity in the hippocampus: regulation of resting membrane potentials by transient receptor potential vanilloid 4.. J Neurosci.

[17] Vriens J, Watanabe H, Janssens A, Droogmans G, Voets T (2004). Cell swelling, heat, and chemical agonists use distinct pathways for the activation of the cation channel TRPV4.. Proc Natl Acad Sci U S A.

[18] Benfenati V, Caprini M, Dovizio M, Mylonakou MN, Ferroni S (2011). An aquaporin-4/transient receptor potential vanilloid 4 (AQP4/TRPV4) complex is essential for cell-volume control in astrocytes.. Proc Natl Acad Sci U S A.

[19] Benfenati V, Ferroni S (2010). Water transport between CNS compartments: functional and molecular interactions between aquaporins and ion channels.. Neuroscience.

[20] Liu X, Bandyopadhyay BC, Nakamoto T, Singh B, Liedtke W (2006). A role for AQP5 in activation of TRPV4 by hypotonicity: concerted involvement of AQP5 and TRPV4 in regulation of cell volume recovery.. J Biol Chem.

[21] Benfenati V, Amiry-Moghaddam M, Caprini M, Mylonakou MN, Rapisarda C (2007). Expression and functional characterization of transient receptor potential vanilloid-related channel 4 (TRPV4) in rat cortical astrocytes.. Neuroscience.

[22] Anderova M, Vorisek I, Pivonkova H, Benesova J, Vargova L (2011). Cell death/proliferation and alterations in glial morphology contribute to changes in diffusivity in the rat hippocampus after hypoxia-ischemia.. J Cereb Blood Flow Metab.

[23] Pivonkova H, Benesova J, Butenko O, Chvatal A, Anderova M (2010). Impact of global cerebral ischemia on K+ channel expression and membrane properties of glial cells in the rat hippocampus.. Neurochem Int.

[24] Makara JK, Rappert A, Matthias K, Steinhauser C, Spat A (2003). Astrocytes from mouse brain slices express ClC-2-mediated Cl- currents regulated during development and after injury.. Mol Cell Neurosci.

[25] Neprasova H, Anderova M, Petrik D, Vargova L, Kubinova S (2007). High extracellular K(+) evokes changes in voltage-dependent K(+) and Na (+) currents and volume regulation in astrocytes.. Pflugers Arch.

[26] Barry PH (1994). JPCalc, a software package for calculating liquid junction potential corrections in patch-clamp, intracellular, epithelial and bilayer measurements and for correcting junction potential measurements.. J Neurosci Methods.

[27] Nimmerjahn A, Kirchhoff F, Kerr JN, Helmchen F (2004). Sulforhodamine 101 as a specific marker of astroglia in the neocortex in vivo.. Nat Methods.

[28] O’Neil RG, Heller S (2005). The mechanosensitive nature of TRPV channels.. Pflugers Arch.

[29] Benesova J, Rusnakova V, Honsa P, Pivonkova H, Dzamba D (2012). Distinct Expression/Function of Potassium and Chloride Channels Contributes to the Diverse Volume Regulation in Cortical Astrocytes of GFAP/EGFP Mice.. PLoS One.

[30] Livak KJ, Schmittgen TD (2001). Analysis of relative gene expression data using real-time quantitative PCR and the 2(-Delta Delta C(T)) Method.. Methods.

[31] Watanabe H, Vriens J, Janssens A, Wondergem R, Droogmans G (2003). Modulation of TRPV4 gating by intra- and extracellular Ca2+.. Cell Calcium.

[32] Vincent F, Acevedo A, Nguyen MT, Dourado M, DeFalco J (2009). Identification and characterization of novel TRPV4 modulators.. Biochem Biophys Res Commun.

[33] Jia Y, Wang X, Varty L, Rizzo CA, Yang R (2004). Functional TRPV4 channels are expressed in human airway smooth muscle cells.. Am J Physiol Lung Cell Mol Physiol.

[34] Zhou M, Kimelberg HK (2000). Freshly isolated astrocytes from rat hippocampus show two distinct current patterns and different [K(+)](o) uptake capabilities.. J Neurophysiol.

[35] Bond CE, Patel P, Crouch L, Tetlow N, Day T (2006). Astroglia up-regulate transcription and secretion of ‘readthrough’ acetylcholinesterase following oxidative stress.. Eur J Neurosci.

[36] Cao X, Zhang Y, Zou L, Xiao H, Chu Y (2010). Persistent oxygen-glucose deprivation induces astrocytic death through two different pathways and calpain-mediated proteolysis of cytoskeletal proteins during astrocytic oncosis.. Neurosci Lett.

[37] Nilius B, Vriens J, Prenen J, Droogmans G, Voets T (2004). TRPV4 calcium entry channel: a paradigm for gating diversity.. Am J Physiol Cell Physiol.

[38] Nett WJ, Oloff SH, McCarthy KD (2002). Hippocampal astrocytes in situ exhibit calcium oscillations that occur independent of neuronal activity.. J Neurophysiol.

[39] Takano T, Oberheim N, Cotrina ML, Nedergaard M (2009). Astrocytes and ischemic injury.. Stroke.

[40] Balazsi G, Cornell-Bell AH, Moss F (2003). Increased phase synchronization of spontaneous calcium oscillations in epileptic human versus normal rat astrocyte cultures.. Chaos.

[41] Manning TJ, Sontheimer H (1997). Spontaneous intracellular calcium oscillations in cortical astrocytes from a patient with intractable childhood epilepsy (Rasmussen’s encephalitis).. Glia.

[42] Krebs C, Fernandes HB, Sheldon C, Raymond LA, Baimbridge KG (2003). Functional NMDA receptor subtype 2B is expressed in astrocytes after ischemia in vivo and anoxia in vitro.. J Neurosci.

[43] Miller B, Sarantis M, Traynelis SF, Attwell D (1992). Potentiation of NMDA receptor currents by arachidonic acid.. Nature.

[44] Meng F, To WK, Gu Y (2008). Role of TRP channels and NCX in mediating hypoxia-induced [Ca(2+)](i) elevation in PC12 cells.. Respir Physiol Neurobiol.

[45] Plant TD, Strotmann R (2007). Trpv4.. Handb Exp Pharmacol.

[46] Zhao X, Gorin FA, Berman RF, Lyeth BG (2008). Differential hippocampal protection when blocking intracellular sodium and calcium entry during traumatic brain injury in rats.. J Neurotrauma.

[47] Kirischuk S, Kettenmann H, Verkhratsky A (1997). Na+/Ca2+ exchanger modulates kainate-triggered Ca2+ signaling in Bergmann glial cells in situ.. FASEB J.

[48] Takuma K, Matsuda T, Hashimoto H, Asano S, Baba A (1994). Cultured rat astrocytes possess Na(+)-Ca2+ exchanger.. Glia.

[49] Kirischuk S, Kettenmann H, Verkhratsky A (2007). Membrane currents and cytoplasmic sodium transients generated by glutamate transport in Bergmann glial cells.. Pflugers Arch.

[50] Rojas H, Colina C, Ramos M, Benaim G, Jaffe EH (2007). Na+ entry via glutamate transporter activates the reverse Na+/Ca2+ exchange and triggers Ca(i)2+-induced Ca2+ release in rat cerebellar Type-1 astrocytes.. J Neurochem.

[51] Paluzzi S, Alloisio S, Zappettini S, Milanese M, Raiteri L (2007). Adult astroglia is competent for Na+/Ca2+ exchanger-operated exocytotic glutamate release triggered by mild depolarization.. J Neurochem.

[52] Bae CY, Sun HS (2011). TRPM7 in cerebral ischemia and potential target for drug development in stroke.. Acta Pharmacol Sin.

[53] Chen HC, Xie J, Zhang Z, Su LT, Yue L (2010). Blockade of TRPM7 channel activity and cell death by inhibitors of 5-lipoxygenase.. PLoS One.

[54] Miller BA, Zhang W (2011). TRP channels as mediators of oxidative stress.. Adv Exp Med Biol.

[55] Runnels LW (2011). TRPM6 and TRPM7: A Mul-TRP-PLIK-cation of channel functions.. Curr Pharm Biotechnol.

[56] Fernandes J, Lorenzo IM, Andrade YN, Garcia-Elias A, Serra SA (2008). IP3 sensitizes TRPV4 channel to the mechano- and osmotransducing messenger 5′-6′-epoxyeicosatrienoic acid.. J Cell Biol.

[57] Fan HC, Zhang X, McNaughton PA (2009). Activation of the TRPV4 ion channel is enhanced by phosphorylation.. J Biol Chem.

[58] Liedtke W (2006). Transient receptor potential vanilloid channels functioning in transduction of osmotic stimuli.. J Endocrinol.

[59] Chvatal A, Anderova M, Hock M, Prajerova I, Neprasova H (2007). Three-dimensional confocal morphometry reveals structural changes in astrocyte morphology in situ.. J Neurosci Res.

[60] Benesova J, Hock M, Butenko O, Prajerova I, Anderova M (2009). Quantification of astrocyte volume changes during ischemia in situ reveals two populations of astrocytes in the cortex of GFAP/EGFP mice.. J Neurosci Res.

[61] Kimelberg HK (2005). Astrocytic swelling in cerebral ischemia as a possible cause of injury and target for therapy.. Glia.

[62] Evanko DS, Zhang Q, Zorec R, Haydon PG (2004). Defining pathways of loss and secretion of chemical messengers from astrocytes.. Glia.

[63] Montana V, Malarkey EB, Verderio C, Matteoli M, Parpura V (2006). Vesicular transmitter release from astrocytes.. Glia.

[64] Volterra A, Meldolesi J (2005). Astrocytes, from brain glue to communication elements: the revolution continues.. Nat Rev Neurosci.

[65] Strotmann R, Schultz G, Plant TD (2003). Ca2+-dependent potentiation of the nonselective cation channel TRPV4 is mediated by a C-terminal calmodulin binding site.. J Biol Chem.

[66] Clemens JA, Stephenson DT, Smalstig EB, Roberts EF, Johnstone EM (1996). Reactive glia express cytosolic phospholipase A2 after transient global forebrain ischemia in the rat.. Stroke.

[67] Rao SP, Sikdar SK (2006). Astrocytes in 17beta-estradiol treated mixed hippocampal cultures show attenuated calcium response to neuronal activity.. Glia.

[68] Hill K, Tigue NJ, Kelsell RE, Benham CD, McNulty S (2006). Characterisation of recombinant rat TRPM2 and a TRPM2-like conductance in cultured rat striatal neurones.. Neuropharmacology.

[69] Voets T, Prenen J, Vriens J, Watanabe H, Janssens A (2002). Molecular determinants of permeation through the cation channel TRPV4.. J Biol Chem.

[70] Rock MJ, Prenen J, Funari VA, Funari TL, Merriman B (2008). Gain-of-function mutations in TRPV4 cause autosomal dominant brachyolmia.. Nat Genet.

[71] Jung C, Fandos C, Lorenzo IM, Plata C, Fernandes J (2009). The progesterone receptor regulates the expression of TRPV4 channel.. Pflugers Arch.

[72] Lanciotti A, Brignone MS, Molinari P, Visentin S, De Nuccio C (2012). Megalencephalic leukoencephalopathy with subcortical cysts protein 1 functionally cooperates with the TRPV4 cation channel to activate the response of astrocytes to osmotic stress: dysregulation by pathological mutations.. Hum Mol Genet.

[73] Strotmann R, Semtner M, Kepura F, Plant TD, Schoneberg T (2010). Interdomain interactions control Ca2+-dependent potentiation in the cation channel TRPV4.. PLoS One.

[74] Arniges M, Fernandez-Fernandez JM, Albrecht N, Schaefer M, Valverde MA (2006). Human TRPV4 channel splice variants revealed a key role of ankyrin domains in multimerization and trafficking.. J Biol Chem.

[75] Kim D (2005). Physiology and pharmacology of two-pore domain potassium channels.. Curr Pharm Des.

[76] Matyash V, Kettenmann H (2010). Heterogeneity in astrocyte morphology and physiology.. Brain Res Rev.

[77] Honsa P, Pivonkova H, Dzamba D, Filipova M, Anderova M (2012). Polydendrocytes Display Large Lineage Plasticity following Focal Cerebral Ischemia.. PLoS One.

[78] Nagelhus EA, Mathiisen TM, Ottersen OP (2004). Aquaporin-4 in the central nervous system: cellular and subcellular distribution and coexpression with KIR4.1.. Neuroscience.

[79] Seifert G, Huttmann K, Binder DK, Hartmann C, Wyczynski A (2009). Analysis of astroglial K+ channel expression in the developing hippocampus reveals a predominant role of the Kir4.1 subunit.. J Neurosci.

[80] Price DL, Ludwig JW, Mi H, Schwarz TL, Ellisman MH (2002). Distribution of rSlo Ca2+-activated K+ channels in rat astrocyte perivascular endfeet.. Brain Res.

[81] Agulhon C, Petravicz J, McMullen AB, Sweger EJ, Minton SK (2008). What is the role of astrocyte calcium in neurophysiology?. Neuron.

[82] Cali C, Marchaland J, Spagnuolo P, Gremion J, Bezzi P (2009). Regulated exocytosis from astrocytes physiological and pathological related aspects.. Int Rev Neurobiol.

