# Supplementary figures and images for: The Increased Activity of TRPV4 Channel in the Astrocytes of the Adult Rat Hippocampus after Cerebral Hypoxia/Ischemia
Source: PLoS One. 2012 Jun 27;7(6):e39959. doi: 10.1371/journal.pone.0039959 (PMC3384594; doi:10.1371/journal.pone.0039959)

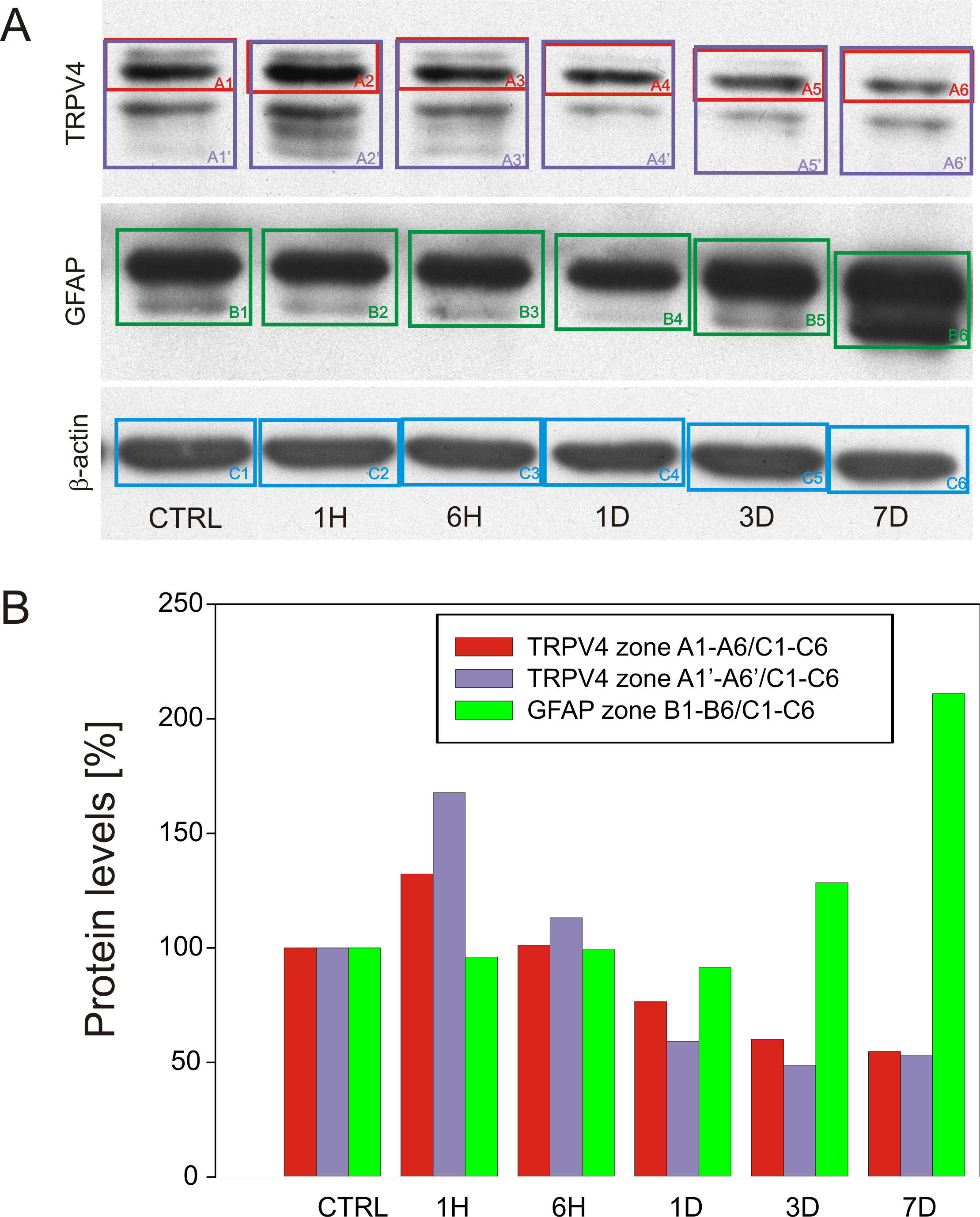

Supplement: Figure S1 — Quantification of TRPV4 protein levels in the hippocampal CA1 region following hypoxia/ischemia. (A) Western blots demonstrating the changes in TRPV4 and GFAP protein levels in response to hypoxic/ischemic (H/I) injury. β-actin was used as a control for sample loading. Squares A1-6 (red) and A1’-6′ (blue) indicate the zones where TRPV4 quantification was carried out, containing 1 or 2 TRPV4 bands, respectively. Squares B1-6 (green) and C1-6 (yellow) indicate the zones where GFAP and β-actin quantification was carried out, respectively. The effect of H/I on TRPV4 levels during the time of reperfusion was evaluated in each individual Western blot and expressed as the percent increase/decrease related to the TRPV4 content in control samples (CTRL), which was set as 100%. To obtain the area corresponding to the TRPV4 protein level at each time-point, the area A1/A1’ (TRPV4) was divided by the area C1 (β-actin) and this value was set as 100%. (B) Single Western blot quantification that demonstrates an increase in TRPV4 protein levels 1H after H/I regardless of the selected zones (A1-6 or A1’-6′) and an increase in GFAP content 3 and 7 days (3D, 7D) after H/I. (TIF) [file pone.0039959.s001.tif]

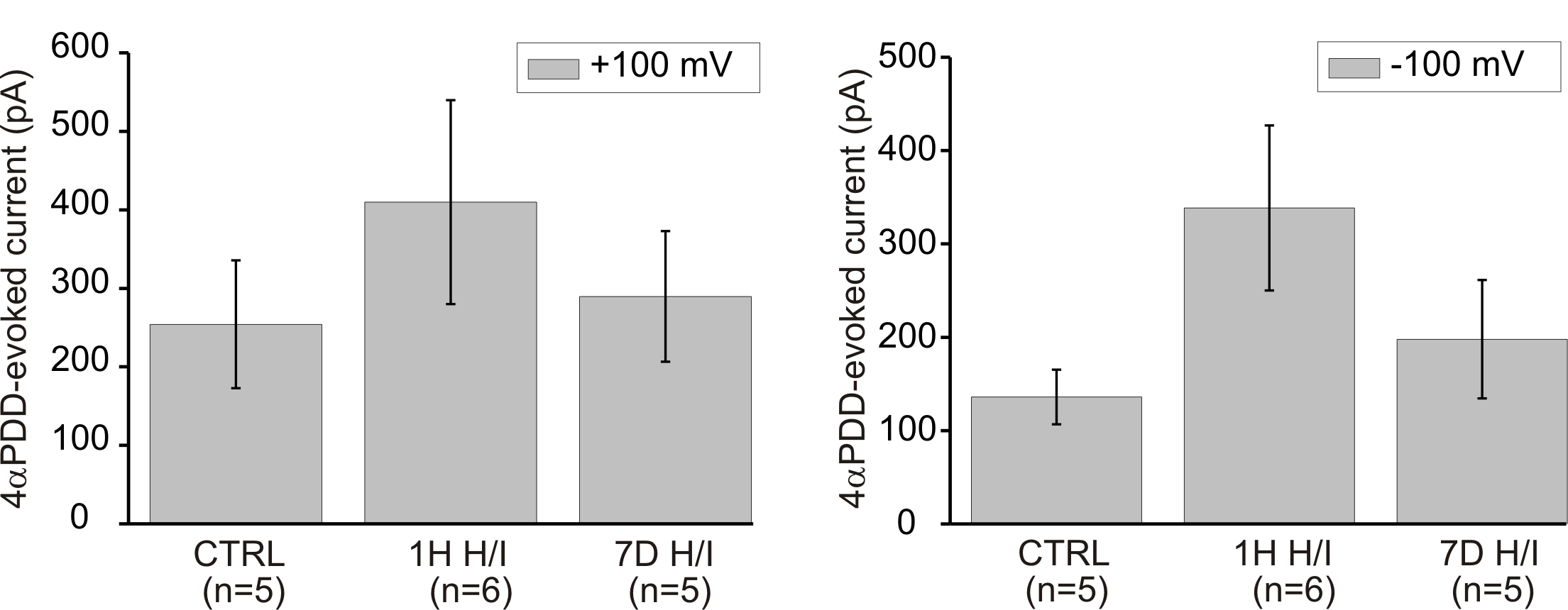

Supplement: Figure S2 — Changes in TRPV4-mediated currents in astrocytes in situ following hypoxia/ischemia. Histograms of 4αPDD-evoked changes in current amplitudes at +100 mV (left) and -100 mV (right) in control astrocytes (CTRL) and astrocytes in slices prepared from rats 1H and 7D after H/I. (TIF) [file pone.0039959.s002.tif]

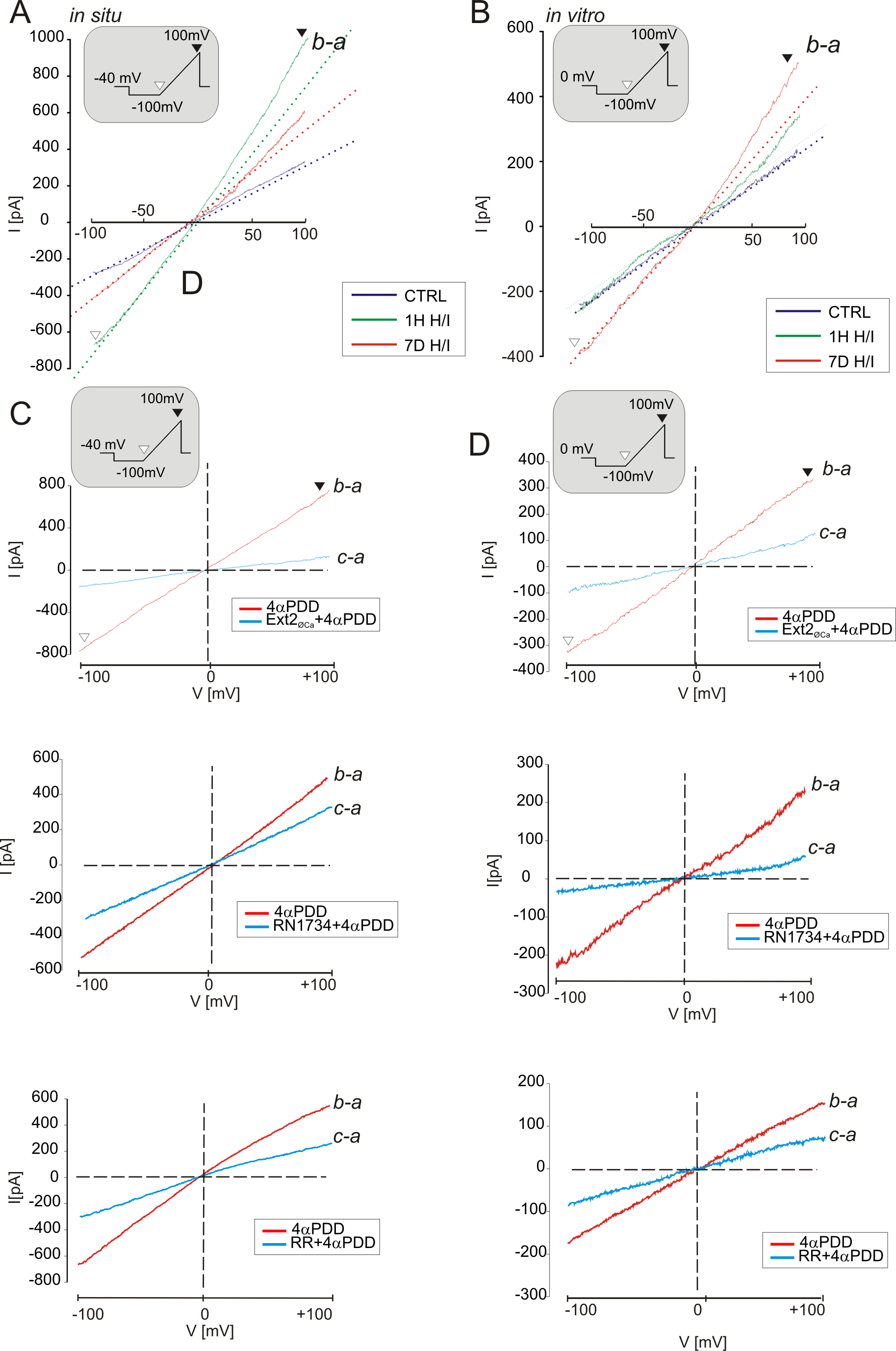

Supplement: Figure S3 — Isolation of 4αPDD currents –point-to-point current subtraction. The 4αPDD- current traces obtained after point-to-point subtraction of ramp current traces recorded prior to 4αPDD application (a) and those recorded during 4αPDD application (b) in astrocytes in situ (A) and astrocytes isolated from the adult rat hippocampal CA1 region (B) of sham-operated rats (CRTL) and rats 1 hour and 7 days after hypoxia/ischemia (1H, 7D H/I; see the voltage ramp protocol in the inset). Note the linear I-V relationship of the 4αPDD-sensitive current (b-a) detected in astrocytes in situ as well as in vitro in sham-operated rats (blue trace), while a modest outward rectification of the 4αPDD current was observed in post-ischemic astrocytes (red and green traces). Typical 4αPDD-current traces obtained after point-to-point subtraction of ramp current traces recorded in astrocytes in situ (C) and astrocytes in vitro (D) prior to (a) and during 4αPDD application (b) and in response to the removal of extracellular Ca2+ (Ext2ØCa, top) or after the application of TRPV4 inhibitors, such as 10 µM RN1734 (middle) or 10 µM,ruthenium red (RR, bottom, c). Red traces represent the 4αPDD-sensitive current (b-a) and blue traces represent the remaining current after applying the inhibitors (c-a). Black and white arrowheads indicate the applied voltage protocol (see the insets) and the corresponding current traces. (TIF) [file pone.0039959.s003.tif]
